# Characterisation of People Living With Chronic Hepatitis B Virus Infection in England and Stratification by HBsAg Levels: A Cross‐Sectional Study

**DOI:** 10.1111/jvh.70101

**Published:** 2025-11-20

**Authors:** Myriam Drysdale, Iain A. Gillespie, Dina Christensen, Jim Davies, Kerrie Woods, Gail Roadknight, Stephanie Little, Hizni Salih, Kinga A. Várnai, Theresa Noble, Graham S. Cooke, Ben Glampson, Dimitri Papadimitriou, Erik Mayer, Salim I. Khakoo, Cai Davis, Florina Borca, Louise English, Eleni Nastouli, Philippa C. Matthews, Eleanor Barnes, Tingyan Wang

**Affiliations:** ^1^ GSK London UK; ^2^ GSK Stevenage UK; ^3^ GSK Upper Providence Pennsylvania USA; ^4^ NIHR Oxford Biomedical Research Centre Oxford UK; ^5^ NIHR Health Informatics Collaborative Oxford University Hospitals NHS Foundation Trust Oxford UK; ^6^ iCARE Secure Data Environment, NIHR Imperial Biomedical Research Centre London UK; ^7^ Faculty of Medicine Imperial College London London UK; ^8^ University Hospitals Southampton NHS Foundation Trust Southampton UK; ^9^ UCL/UCLH NIHR Biomedical Research Centre University College London Hospitals NHS Foundation Trust London UK; ^10^ The Francis Crick Institute London UK; ^11^ Nuffield Department of Medicine University of Oxford Oxford UK

**Keywords:** chronic hepatitis B, cross‐sectional studies, hepatitis B surface antigens

## Abstract

Characterisation of people with hepatitis B (PwHB) remains limited, particularly regarding treatment status, disease severity and biomarker profiles. Quantitative hepatitis B surface antigen (qHBsAg) is a key predictor of response to emerging therapies, but its distribution is poorly described. Using a large, ethnically diverse UK cohort, we assessed demographics, clinical features and HBsAg levels to guide treatment strategies. This cross‐sectional analysis of PwHB (*N* = 2000 [prespecified]) used data from four English hospitals, collected via the National Institute for Health and Care Research Health Informatics Collaborative framework. Individual characteristics were assessed overall, and *post hoc* by qHBsAg levels (≤ 3000/> 3000 IU/mL; < 100/≥ 100–≤ 1000/> 1000 IU/mL) available from one centre (*N* = 457). The cohort had a slight male predominance (54%) and a mean age of 44.9 years. White and Asian ethnicity each accounted for 25%, and 23% were on nucleos(t)ide analogue therapy. Centres collecting HBsAg data had more individuals with undetectable HBV DNA or on treatment. Among individuals with non‐missing qHBsAg data (263/457), 167/263 (63.5%) had qHBsAg ≤ 3000 IU/mL. These were older (49.6 vs. 43.5 years), more likely to be male (53.9% vs. 35.4%), Asian (40.7% vs. 20.8%) or have undetectable HBV DNA (35.9% vs. 17.7%), and less likely to be Black (13.2% vs. 34.4%) versus those with qHBsAg > 3000 IU/mL. Fifty‐six (21.3%) people had qHBsAg < 100 IU/mL, 60 (22.8%) between ≥ 100 and ≤ 1000 IU/mL, and 147 (55.9%) > 1000 IU/mL. This cohort of PwHB highlights qHBsAg distribution in clinical settings and could identify people more likely to achieve functional cure with emerging therapies.

**Abbreviations**
ALT: alanine aminotransferase
CKD: chronic kidney disease
EHR: electronic health record
HBeAg: hepatitis B e‐antigen
HBsAg: hepatitis B surface antigen
HBV: hepatitis B virus
HCC: hepatocellular carcinoma
HIC: Health Informatics Collaborative
IFN: interferon
IQR: interquartile range
NA: nucleos(t)ide analogue
NICE: National Institute for Health and Care Excellence
NIHR: National Institute for Health and Care Research
qHBsAg: quantitative hepatitis B surface antigen
S/CO: signal‐to‐cut‐off ratio
SD: standard deviation

Chronic hepatitis B virus (HBV) infection is a liver disease of major public health significance [1, 2, 3]. In 2022, an estimated 257.5 million individuals were living with HBV infection globally [4]. In the same year, there were approximately 270,000 people living with hepatitis B in the UK, accounting for 0.6% of the population [5], despite high infant vaccination coverage (91.7% as of December 2023 for the hexavalent vaccine, which includes HBV, introduced in 2017) [6]. An estimated 95% of new diagnoses for chronic HBV infections in England typically occur in those who have acquired the infection in early life in endemic regions [7].

The natural history of chronic HBV infection is dynamic and characterized by distinct patterns of serologic markers, HBV DNA and alanine aminotransferase (ALT) levels, as well as the immunological and hepatic neuroinflammatory status [8, 9]. Heterogeneity in progression contributes to the development of key long‐term complications, including cirrhosis, liver decompensation and hepatocellular carcinoma (HCC) [8]. Chronic HBV treatment seeks to reduce the risk of disease progression, thereby improving survival and quality of life, which can be measured through surrogate disease markers (e.g., ALT, HBV DNA, hepatitis B e antigen [HBeAg]) [8]. Clinical guidelines for the management of chronic HBV infection often provide treatment advice on the basis of these parameters [10, 11, 12], but only an estimated 31% of treatment‐eligible people in the UK receive treatment [4]. Consequently there is a need for greater real‐world insights into the characteristics of people living with chronic HBV infection with increasing levels of disease progression, both treated and untreated. More broadly, and because of the interplay between ethnicity, infecting HBV genotype and disease progression [13], there is value in understanding biomarker distribution by ethnicity.

Hepatitis B surface antigen (HBsAg) presence is a hallmark of HBV infection and is linked to chronicity, as high levels contribute to immune exhaustion and viral persistence [14, 15]. HBsAg loss is associated with significantly reduced risks of HCC and decompensated liver disease versus HBV DNA suppression alone, underscoring its importance as part of the optimal treatment endpoint (functional cure) in disease management [15, 16, 17]. Furthermore, baseline HBsAg levels are an important predictor of HBsAg loss and a positive response to treatment [15, 18, 19]. However, as HBsAg loss is uncommon (on‐ or off‐treatment) [20] and the therapeutic management of people with chronic HBV infection is not currently influenced by HBsAg levels, most clinical laboratories do not measure HBsAg, or perform qualitative (semi‐quantitative) rather than fully quantitative assays. Until recently [9], quantitative HBsAg (qHBsAg) testing was not included in clinical guidelines [10, 21]. As a result, there are limited data on qHBsAg in the literature [22].

qHBsAg measurement is increasingly recognised for its role in managing chronic HBV infection. In the UK, the National Institute for Health and Care Excellence (NICE) advises monitoring HBV DNA and qHBsAg levels, along with HBeAg status, before initiating treatment and at specified intervals thereafter to assess treatment response and adherence [12]. The updated EASL 2025 guidelines also support this approach [9]. Furthermore, novel HBV therapies are being developed for people with a specific HBsAg quantification cut‐off [23, 24], as lower HBsAg levels have been associated with improved response [19, 25].

Utilising a large, ethnically diverse national cohort, this study aimed to describe the demographic, clinical and treatment characteristics in people living with chronic HBV infection in England. Individuals were originally stratified by HBeAg, ethnicity, treatment and liver disease status; with increasing interest in qHBsAg levels, a post hoc analysis was conducted to stratify our cohort by this parameter, which is the focus of this research.

## 1 Methods

### 1.1 Study Design and Data Source

This descriptive cross‐sectional study utilised the National Institute for Health and Care Research (NIHR) Health Informatics Collaborative (HIC) Viral Hepatitis and Liver Disease Theme Database [26, 27]. Curated electronic health record (EHR) data were obtained from a pre‐specified number of randomly selected individuals (*N* = 2000) from four NHS trusts contributing to the NIHR HIC Viral Hepatitis database: Imperial College Healthcare NHS Trust, Oxford University Hospitals NHS Foundation Trust, University College London Hospitals NHS Foundation Trust, and University Hospitals Southampton NHS Foundation Trust (Figure S1). In accordance with the data sharing agreement, the names of these centres have been anonymised (referred to as Centres 1 to 4 throughout the text).

### 1.2 Eligibility Criteria, Study Population and Study Period

The study cohort comprised people with chronic HBV infection from the collaborating centres, who were identified using codes based on the results of HBsAg and HBV DNA tests between 1 January 2016 and 31 December 2019 (identification period; Figure S2). The first evidence of chronic HBV infection (defined as two positive HBsAg tests 6 months apart or a positive HBsAg plus an HBV DNA test 6 months apart) within the identification period defined the index date. People were excluded if they had acute HBV infection or a single HBsAg test which could not be conclusively proven to be chronic (for complete inclusion/exclusion criteria see Table S1). Baseline demographic and clinical characteristics were assessed over a 365‐day period prior to the disease index date. In total, 2925 individuals met the pre‐defined eligibility criteria. In accordance with the study protocol, which stipulated the inclusion of 2000 individuals, a stratified selection approach was implemented to preserve population heterogeneity and ensure balanced representation across sites. All eligible individuals from three centres (Centres 1–3) with smaller sample sizes were included in the final analysis. To reach the target sample size, the remaining individuals were randomly selected from the centre (Centre 4) with the largest number of eligible individuals (Table S2).

### 1.3 Variables and Assessments

Demographics, clinical characteristics and laboratory measurements were collected, with baseline values used for all variables (for laboratory measurements, the last value within the baseline period was used; age was recorded at the index date). Variables included in the analysis are detailed in Table S3; data were stratified by self‐reported ethnicity [28] (using standard national codes in the UK [29]: White, Mixed, Asian, Black, other/unknown), liver disease status (no evidence of liver disease, any evidence of liver disease of increasing severity [none < fibrosis < compensated cirrhosis < decompensated cirrhosis < HCC]), treatment (no treatment, nucleos[t]ide analogue [NA] monotherapy/combinations), HBeAg status (positive, negative, missing), and qHBsAg levels (described below).

### 1.4 HBsAg Levels

Two centres routinely assessed HBsAg levels. Centre 1 measured blood qHBsAg levels using the fully quantitative Architect HBsAg QT (Abbott Corp, Illinois, US), reported in IU/mL. Two stratifications, commonly used in clinical development programmes of new therapeutic agents, were used for these data (broad: ≤ 3000 IU/mL, > 3000 IU/mL or missing; granular: < 100 IU/mL, ≥ 100–1000 IU/mL, > 1000 IU/mL, or missing). Centre 2 used the Architect HBsAg Qualitative II assay to report semi‐quantitative HBsAg levels, expressed as a signal‐to‐cut‐off ratio (S/CO) or index value. S/CO < 1 indicated a negative result for HBsAg, whilst S/CO ≥ 1 indicated a positive result for HBsAg, with higher values suggesting higher HBsAg levels. Two stratifications were performed on HBsAg semi‐quantitative data from Centre 2 (a broad one using the median value alone as a cut‐off, and a more granular assessment that included S/CO < 100 and the median value).

### 1.5 Data Analysis

Categorical data were analyzed using frequencies and percentages. For outcome variables, 95% confidence intervals (CIs) were calculated, with exact CIs reported when:

$$\text{proportion}\times \text{denominator}<10,\text{or}\;\left(1-\text{proportion}\right)\times \text{denominator}<10.$$

Continuous data were summarized as means ± standard deviation (SD), ranges, and medians with interquartile ranges (IQR). The analysis was stratified according to the variables outlined above; qHBsAg analyses were conducted post hoc.

### 1.6 Ethical Considerations

This project was approved by the National Research Ethics Service Committee South Central–Oxford on 26 February 2021 (REC reference 21/SC/0060). This study complied with all applicable laws regarding subject privacy. No direct subject contact or primary collection of individual human subject data occurred. Results are presented in tabular form as aggregate analyses that preclude subject identification.

## 2 Results

### 2.1 Overall Study Cohort

Overall, 2000 adults living with chronic HBV infection were included for analysis across the four contributing NHS Trusts in England (Figure S1). The mean (SD) age of the overall cohort was 44.9 (12.7) years, with 54% being male (Table 1). White and Asian ethnicity each accounted for a quarter of the population. Most people were HBeAg negative with no evidence of viral co‐infections, liver disease, or chronic kidney disease (CKD). Approximately 23% of people were treated with either NA monotherapy or combination therapy.

**TABLE 1:** Characteristics of the overall study cohort and by qHBsAg testing status: Centres 1 and 2 (qHBsAg and semi‐quantitative HBsAg) versus Centres 3 and 4 (non‐testing).

Variable	Overall (*N* = 2000)		Centres that collect qHBsAg or semi‐quantitative HBsAg data (Centres 1 and 2) (*n* = 1095)		Centres that do not collect qHBsAg or semi‐quantitative HBsAg data (Centres 3 and 4) (*n* = 905)	
	*n* (%)	95% CI	*n* (%)	95% CI	*n* (%)	95% CI
Male sex	1079 (54)	51.8–56.1	587 (53.6)	50.7–56.6	492 (54.4)	51.1–57.6
Age group, years						
< 25	48 (2.4)	1.7–3.1	23 (2.1)	1.3–2.9	25 (2.8)	1.7–3.8
25–49	1321 (66.1)	64–68.1	743 (67.9)	65.1–70.6	578 (63.9)	60.7–67.0
50–64	467 (23.4)	21.5–25.2	242 (22.1)	19.6–24.6	225 (24.9)	22.0–27.7
≥ 65	164 (8.2)	7.0–9.4	87 (7.9)	6.3–9.5	77 (8.5)	6.7–10.3
Ethnicity^a^						
White	495 (24.8)	22.9–26.6	291 (26.6)	24.0–29.2	204 (22.5)	19.8–25.3
Mixed	62 (3.1)	2.3–3.9	42 (3.8)	2.7–5.0	20 (2.2)	1.3–3.2
Asian	500 (25)	23.1–26.9	300 (27.4)	24.8–30.0	200 (22.1)	19.4–24.8
Black	383 (19.2)	17.4–20.9	194 (17.7)	15.5–20.0	189 (20.9)	18.2–23.5
Other/unknown	560 (28)	26.0–30.0	268 (24.5)	21.9–27.0	292 (32.3)	29.2–35.3
Co‐infections (HIV and/or HCV)	38 (1.9)	1.3–2.5	25 (2.3)	1.4–3.2	13 (1.4)	0.7–2.2
Hepatitis delta	6 (0.3)	0.1–0.7	6 (0.5)	0.2–1.2	0	
Liver fibrosis	89 (4.5)	3.5–5.4	36 (3.3)	2.2–4.3	53 (5.9)	4.3–7.4
Cirrhosis						
Compensated	137 (6.9)	5.7–8.0	43 (3.9)	2.8–5.1	94 (10.4)	8.4–12.4
Decompensated	18 (0.9)	0.5–1.3	14 (1.3)	0.6–1.9	4 (0.4)	0.1–1.1
HCC	12 (0.6)	0.3–0.9	6 (0.5)	0.2–1.2	6 (0.7)	0.2–1.4
CKD	104 (5.2)	4.2–6.2	51 (4.7)	3.4–5.9	53 (5.9)	4.3–7.4
HBeAg status						
Positive	133 (9.4)	7.9–11.0	80 (9.0)	7.1–10.9	53 (10.2)	7.6–12.8
Negative	1278 (90.6)	89.0–92.1	811 (91.0)	89.1–92.9	467 (89.8)	87.2–92.4
Missing	589 (29.5)	27.5–31.4	204 (18.6)	16.3–20.9	385 (42.5)	39.3–45.8
HBV DNA, IU/mL						
Undetectable	230 (11.8)	10.4–13.3	206 (19.6)	17.2–22.0	24 (2.7)	1.6–3.8
Detectable— < 2000	1455 (75.0)	73.0–76.9	741 (70.4)	67.6–73.1	714 (80.4)	77.8–83.0
2000– < 20,000	182 (9.4)	8.1–10.7	76 (7.2)	5.7–8.8	106 (11.9)	9.8–14.1
≥ 20,000	74 (3.8)	3.0–4.7	30 (2.8)	1.8–3.9	44 (5.0)	3.5–6.4
Missing	59 (3.0)	2.2–3.7	42 (3.8)	2.7–5.0	17 (1.9)	1.0–2.8
ALT						
≤ ULN	1665 (84.6)	83.0–86.2	950 (88.5)	86.6–90.4	715 (79.8)	77.2–82.4
1– < 2 × ULN	239 (12.1)	10.7–13.6	102 (9.5)	7.8–11.3	137 (15.3)	12.9–17.6
2– < 5 × ULN	55 (2.8)	2.1–3.5	15 (1.4)	0.7–2.1	40 (4.5)	3.1–5.8
≥ 5 × ULN	10 (0.5)	0.2–0.8	6 (0.6)	0.2–1.2	4 (0.4)	0.01–1.1
Missing	31 (1.6)	1.0–2.1	22 (2.0)	1.2–2.8	9 (1.0)	0.5–1.9
Treatment						
NA monotherapy	435 (21.8)	19.9–23.6	383 (35.0)	32.2–37.8	52 (5.7)	4.2–7.3
NA combination therapy	27 (1.4)	0.8–1.9	19 (1.7)	1–2.5	8 (0.9)	0.4–1.7
Prior interferon use	2 (0.1)	0.0–0.4	0		2 (0.2)	0.03–0.8
No treatment	1538 (76.9)	75.1–78.7	693 (63.3)	60.4–66.1	845 (93.4)	91.7–95.0

*Note:* The table shows descriptive baseline data only; no formal hypothesis testing was conducted.

Abbreviations: ALT, alanine aminotransferase; CI, confidence interval; CKD, chronic kidney disease; HBeAg, hepatitis B e antigen; HBsAg, hepatitis B surface antigen; HBV, hepatitis B virus; HCC, hepatocellular carcinoma; HCV, hepatitis C virus; HIV, human immunodeficiency virus; NA, nucleos(t)ide analogue; qHBsAg, quantitative hepatitis B surface antigen; ULN, upper limit of normal.

^a^ White = ‘White—British’, ‘White—Irish’, ‘White—any other White background’; Asian = ‘Asian or Asian British—Indian Asian or Asian British—Pakistani’, ‘Asian or Asian British—Bangladeshi’, ‘Asian or Asian British—any other Asian background’, ‘Chinese’; Black = ‘Black or Black British—Caribbean’, ‘Black or Black British—African’, ‘Black or Black British—any other Black background’; Mixed = ‘Mixed—White and Black Caribbean’, ‘Mixed—White and Black African’, ‘Mixed—White and Asian/Mixed—any other mixed background'; Other = ‘any other ethnic group’. Ethnicity was self‐reported by individuals and recorded using standard national codes in the UK.

### 2.2 Stratification by Self‐Reported Ethnicity

Demographic and clinical characteristics varied by ethnicity across the four centres (Table 2). Sex and age distribution were similar across ethnicities, although fewer people of Black ethnicity were aged ≥ 65 years (5.5% [95% CI: 3.2–7.8]) versus Asian (10.6% [7.9–13.3]) and White (10.3% [7.6–13.0]) people. Where laboratory data were available, Asian people were more often HBeAg‐positive (14.1% [10.5–17.7]) compared with their White (7.6% [4.8–10.4]) and Black (4.4% [2.0–6.9]) counterparts. Excluding people reporting other/unknown ethnicity, the highest proportion of untreated people was observed in the Black stratum (80.4%), followed by the White (75.8%) and Asian (69.6%) strata. CKD was reported more frequently in Asian (7.2% [4.9–9.5]) and Black (7.8% [5.1–10.5]) than White people (3.4% [1.8–5.0]).

**TABLE 2:** Characteristics of the study cohort by self‐reported ethnicity.^a^

Variable	White (*n* = 495)		Mixed (*n* = 62)		Asian (*n* = 500)		Black (*n* = 383)		Other/unknown (*n* = 560)	
	*n* (%)	95% CI	*n* (%)	95% CI	*n* (%)	95% CI	*n* (%)	95% CI	*n* (%)	95% CI
Male sex	266 (53.7)	49.3–58.1	35 (56.5)	44.1–68.8	254 (50.8)	46.4–55.2	203 (53.0)	48.0–58.0	321 (57.3)	53.2–61.4
Age, years										
Mean (SD)	45.4 (12.9)		44.9 (11.8)		46.3 (13.5)		45.3 (11.4)		43.0 (12.8)	
Median (IQR)	43.0 (35.0–53.0)		44.0 (35.0–52.5)		44.0 (37.0–56.0)		45.0 (37.0–52.0)		42.0 (34.0–50.0)	
Age group, years										
< 25	5 (1.0)	0.3–2.3	0		9 (1.8)	0.8–3.4	8 (2.1)	0.9–4.1	26 (4.6)	2.9–6.3
25–49	328 (66.3)	62.1–70.4	45 (72.6)	61.5–83.7	312 (62.4)	58.2–66.6	250 (65.3)	60.5–70.0	386 (68.9)	65.1–72.8
50–64	111 (22.4)	18.7–26.1	15 (24.2)	13.5–34.9	126 (25.2)	21.4–29	104 (27.2)	22.7–31.6	111 (19.8)	16.5–23.1
≥ 65	51 (10.3)	7.6–13	2 (3.2)	0.4–11.2	53 (10.6)	7.9–13.3	21 (5.5)	3.2–7.8	37 (6.6)	4.5–8.7
Co‐infections (HIV and/or HCV)	12 (2.4)	1.1–3.7	2 (3.2)	0.4–11.2	2 (0.4)	0.1–1.4	14 (3.7)	1.8–5.6	8 (1.4)	0.6–2.8
Hepatitis delta	4 (0.8)	0.2–2.1	0		0		0		2 (0.4)	0–1.3
Liver fibrosis	18 (3.6)	2.0–5.2	4 (6.5)	1.8–15.8	22 (4.4)	2.6–6.2	15 (3.9)	2.0–5.8	30 (5.4)	3.5–7.3
Cirrhosis (any history)										
Compensated	30 (6.1)	4.0–8.2	6 (9.7)	3.6–19.9	36 (7.2)	4.9–9.5	25 (6.5)	4.0–9.0	40 (7.1)	5.0–9.2
Decompensated	9 (1.8)	0.8–3.4	0		6 (1.2)	0.4–2.6	3 (0.8)	0.2–2.3	0	
HCC (any history)	6 (1.2)	0.4–2.6	0		1 (0.2)	0–1.1	2 (0.5)	0.1–1.9	3 (0.5)	0.1–1.6
CKD	17 (3.4)	1.8–5.0	1 (1.6)	0–8.7	36 (7.2)	4.9–9.5	30 (7.8)	5.1–10.5	20 (3.6)	2.0–5.1
qHBsAg	(*n* = 71)		(*n* = 4)		(*n* = 88)		(*n* = 55)		(*n* = 45)	
Mean (SD)	5523.4 (10,104.8)		2292.3 (4193.9)		5871.4 (23,538.7)		8280.7 (9652.0)		6470.8 (16,799.2)	
Median (IQR)	1201 (100.5–6681.8)		300.2 (0–2592.5)		763.9 (120.8–2679.9)		4462.7 (1368.7–11,820.1)		1749.2 (402.3–5304.3)	
qHBsAg broad stratification										
≤ 3000 IU/mL	46 (64.8)	53.7–75.9	3 (75.0)	19.4–99.4	68 (77.3)	68.5–86.0	22 (40.0)	27.1–52.9	28 (62.2)	48.1–76.4
> 3000 IU/mL	25 (35.2)	24.1–46.3	1 (25.0)	0.6–80.6	20 (22.7)	14.0–31.5	33 (60.0)	47.1–72.9	17 (37.8)	23.6–51.9
Missing (Centre 1)	48 (40.3)	31.5–49.2	12 (75.0)	47.6–92.7	50 (36.2)	28.2–44.3	52 (48.6)	39.1–58.1	32 (41.6)	30.6–52.6
Missing (all centres)	424 (85.7)	82.6–88.7	58 (93.5)	84.3–98.2	412 (82.4)	79.1–85.7	328 (85.6)	82.1–89.2	515 (92.0)	89.7–94.2
qHBsAg granular stratification										
< 100 IU/mL	18 (25.4)	15.2–35.5	2 (50.0)	6.8–93.2	21 (23.9)	15.0–32.8	6 (10.9)	4.1–22.2	9 (20.0)	9.6–34.6
≥ 100 and ≤ 1000 IU/mL	16 (22.5)	12.8–32.3	1 (25.0)	0.6–80.6	30 (34.1)	24.2–44.0	6 (10.9)	4.1–22.2	7 (15.6)	6.5–29.5
> 1000 IU/mL	37 (52.1)	40.5–63.7	1 (25.0)	0.6–80.6	37 (42.0)	31.7–52.4	43 (78.2)	67.3–89.1	29 (64.4)	50.5–78.4
Missing (Centre 1)	48 (40.3)	31.5–49.2	12 (75.0)	47.6–92.7	50 (36.2)	28.2–44.3	52 (48.6)	39.1–58.1	32 (41.6)	30.6–52.6
Missing (all centres)	424 (85.7)	82.6–88.7	58 (93.5)	84.3–98.2	412 (82.4)	79.1–85.7	328 (85.6)	82.1–89.2	515 (92.0)	89.7–94.2
HBeAg status										
Positive	26 (7.6)	4.8–10.4	6 (13.6)	5.2–27.4	51 (14.1)	10.5–17.7	12 (4.4)	2.0–6.9	38 (9.7)	6.8–12.7
Negative	318 (92.4)	89.6–95.2	38 (86.4)	72.6–94.8	311 (85.9)	82.3–89.5	259 (95.6)	93.1–98.0	352 (90.3)	87.3–93.2
Missing	151 (30.5)	26.4–34.6	18 (29.0)	17.7–40.3	138 (27.6)	23.7–31.5	112 (29.2)	24.7–33.8	170 (30.4)	26.5–34.2
HBV DNA, IU/mL										
Undetectable	76 (15.8)	12.6–19.1	10 (16.4)	7.1–25.7	81 (16.7)	13.4–20.1	29 (7.8)	5.1–10.5	34 (6.3)	4.2–8.3
Detectable— < 2000	348 (72.5)	68.5–76.5	44 (72.1)	60.9–83.4	352 (72.7)	68.8–76.7	292 (78.3)	74.1–82.5	419 (77.2)	73.6–80.7
2000– < 20,000	43 (9.0)	6.4–11.5	4 (6.6)	1.8–15.9	32 (6.6)	4.4–8.8	43 (11.5)	8.3–14.8	60 (11.0)	8.4–13.7
≥ 20,000	13 (2.7)	1.3–4.2	3 (4.9)	1.0–13.7	19 (3.9)	2.2–5.7	9 (2.4)	1.1–4.5	30 (5.5)	3.6–7.4
Missing	15 (3.0)	1.5–4.5	1 (1.6)	0–8.7	16 (3.2)	1.7–4.7	10 (2.6)	1.0–4.2	17 (3.0)	1.6–4.5
ALT										
≤ ULN	414 (85.0)	81.8–88.2	52 (88.1)	77.1–95.1	419 (85.0)	81.8–88.1	331 (88.0)	84.8–91.3	449 (81.0)	77.8–84.3
1– < 2 × ULN	56 (11.5)	8.7–14.3	5 (8.5)	2.8–18.7	60 (12.2)	9.3–15.1	38 (10.1)	7.1–13.2	80 (14.4)	11.5–17.4
2– < 5 × ULN	13 (2.7)	1.2–4.1	2 (3.4)	0.4–11.7	12 (2.4)	1.1–3.8	6 (1.6)	0.6–3.4	22 (4.0)	2.3–5.6
≥ 5 × ULN	4 (0.8)	0.2–2.1	0		2 (0.4)	0–1.5	1 (0.3)	0–1.5	3 (0.5)	0.1–1.6
Missing	8 (1.6)	0.7–3.2	3 (4.8)	1.0–13.5	7 (1.4)	0.6–2.9	7 (1.8)	0.7–3.7	6 (1.1)	0.4–2.3
Treatment										
NA monotherapy	111 (22.4)	18.7–26.1	14 (22.6)	12.2–33.0	144 (28.8)	24.8–32.8	72 (18.8)	14.9–22.7	94 (16.8)	13.7–19.9
NA combination therapy	9 (1.8)	0.8–3.4	2 (3.2)	0.4–11.2	8 (1.6)	0.7–3.1	3 (0.8)	0.2–2.3	5 (0.9)	0.3–2.1
Prior interferon use	1 (0.2)	0–1.1	0		1 (0.2)	0–1.1	0		0	
No treatment	375 (75.8)	72–79.5	46 (74.2)	63.3–85.1	348 (69.6)	65.6–73.6	308 (80.4)	76.4–84.4	461 (82.3)	79.2–85.5

*Note:* The table shows descriptive baseline data only; no formal hypothesis testing was conducted.

Abbreviations: ALT, alanine aminotransferase; CI, confidence interval; CKD, chronic kidney disease; HBeAg, hepatitis B e antigen; HBsAg, hepatitis B surface antigen; HBV, hepatitis B virus; HCC, hepatocellular carcinoma; HCV, hepatitis C virus; HIV, human immunodeficiency virus; IQR, interquartile range; NA, nucleos(t)ide analogue; qHBsAg, quantitative HBsAg; SD, standard deviation; ULN, upper limit of normal.

^a^ White = ‘White—British’, ‘White—Irish’, ‘White—any other White background’; Asian = ‘Asian or Asian British—Indian Asian or Asian British—Pakistani’, ‘Asian or Asian British—Bangladeshi’, ‘Asian or Asian British—any other Asian background’, ‘Chinese’; Black = ‘Black or Black British—Caribbean’, ‘Black or Black British—African’, ‘Black or Black British—any other Black background’; Mixed = ‘Mixed—White and Black Caribbean’, ‘Mixed—White and Black African’, ‘Mixed—White and Asian/Mixed—any other mixed background'; Other = ‘any other ethnic group’. Ethnicity was self‐reported by individuals and recorded using standard national codes in the UK.

### 2.3 Stratification by Liver Disease, Treatment and HBeAg

Across the four centres, people with evidence of liver disease (*n* = 191) were more likely to be aged ≥ 50 years (56.5% [95% CI: 49.5–63.6] vs. 28.9% [26.8–31.0]), have CKD (16.2% [11.0–21.5] vs. 4.0% [3.1–4.9]), be co‐infected with human immunodeficiency virus or hepatitis C virus (5.2% [2.5–9.4] vs. 1.5% [0.9–2.1]) and be HBeAg‐positive (16.8% [10.2–23.4] vs. 8.7% [7.2–10.3]) than those without liver disease (Table S4).

Approximately three‐quarters of people received no treatment for chronic HBV infection, but among those receiving treatment (23%, *N* = 462), 435 and 27 had NA monotherapy (primarily tenofovir) and NA combination therapy, respectively (Table S4). No individuals were receiving interferon (IFN) at the index date, although two people had a record of prior IFN treatment. People receiving NAs were more likely to be male (63.6% [59.2–68.0]) versus those untreated (51.0% [48.5–53.5]) (Table S5). Treated people were also older than untreated ones, with higher proportions in the 50–64 years (31.0% [26.7–35.2] vs. 21.1% [19.0–23.1]) and 65+ years (13.0% [9.9–16.1] vs. 6.8% [5.5–8.0]) age groups. Most people had undetectable or low (< 2000 IU/mL) HBV DNA levels (92.6% [428/462] of people receiving NA treatment and 81.7% [1257/1538] of people receiving no treatment; Table S5).

HBeAg data were missing for 589 (29.5%) individuals across the four centres; among those with HBeAg data, 90.6% (1278/1411) were HBeAg‐negative (Table S6). HBeAg‐positive status was mainly reported in younger adults. As expected, HBeAg‐positive status was observed in people with higher HBV DNA levels. Characteristics of people with missing HBeAg data were generally similar to those of HBeAg‐negative people, suggesting that clinicians may stop testing for HBeAg after anti‐HBe seroconversion.

Characteristics were largely similar between centres collecting HBsAg data and those that did not (Table 1). Notable differences were that the proportions of people with undetectable HBV DNA, and the proportions of people receiving treatment, were both higher in centres collecting HBsAg data than in those that did not.

### 2.4 Stratification by qHBsAg Test Result Availability (in Centres Routinely Testing for HBsAg)

In the centres that routinely measured HBsAg levels (Centres 1 and 2), the demographic characteristics of people with complete HBsAg data were generally similar to those with missing HBsAg data (Table S7). There was a greater proportion of Black people in the stratum with missing (27.0% [95% CI: 21.3–32.7]) versus complete (15.2% [12.8–17.6]) HBsAg data. With respect to laboratory values and clinical characteristics, the proportion of people with undetectable HBV DNA data was higher among those with complete versus missing HBsAg data (21.5% [18.7–24.3] vs. 12.7% [8.4–17.0]). This was coincident with treatment, with more people with complete HBsAg data treated with NA monotherapy than those with missing HBsAg data (37.4% [34.1–40.6] vs. 26.2% [20.5–31.8]).

### 2.5 Stratification by qHBsAg Levels (in Centres Routinely Testing for HBsAg)

**FIGURE 1:**
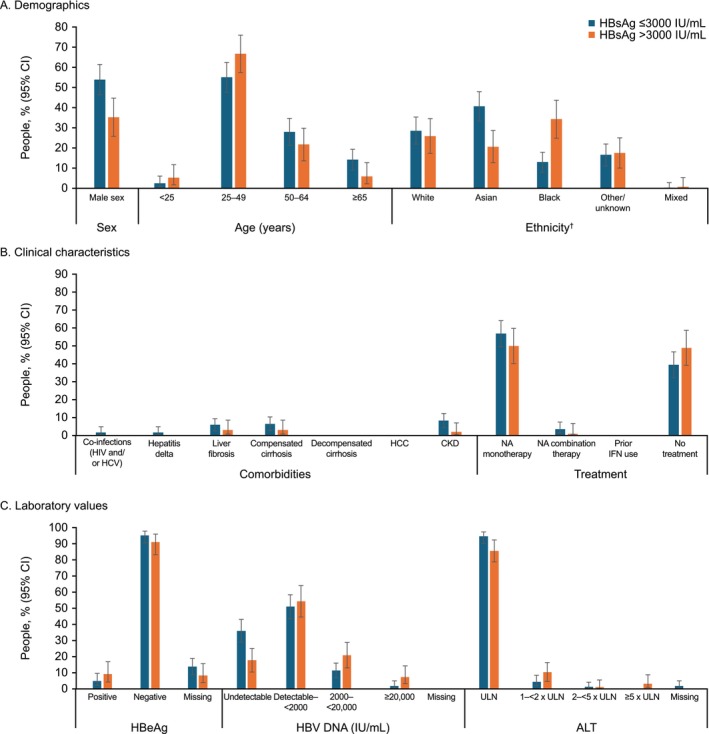
Characteristics of the study cohort by broad qHBsAg stratification: (A) demographics, (B) clinical characteristics and (C) laboratory values (Centre 1). White = ‘White—British’, ‘White—Irish’, ‘White—any other White background’; Asian = ‘Asian or Asian British—Indian Asian or Asian British—Pakistani’, ‘Asian or Asian British—Bangladeshi’, ‘Asian or Asian British—any other Asian background’, ‘Chinese’; Black = ‘Black or Black British—Caribbean’, ‘Black or Black British—African’, ‘Black or Black British—any other Black background’; Mixed = ‘Mixed—White and Black Caribbean’, ‘Mixed—White and Black African’, ‘Mixed—White and Asian/Mixed—any other mixed background'; Other = ‘any other ethnic group’. Ethnicity was self‐reported by individuals and recorded using standard national codes in the UK. ALT, alanine aminotransferase; CI, confidence interval; CKD, chronic kidney disease; HBeAg, hepatitis B e antigen; HBsAg, hepatitis B surface antigen; HBV, hepatitis B virus; HCC, hepatocellular carcinoma; HCV, hepatitis C virus; HIV, human immunodeficiency virus; IFN, interferon; NA, nucleos(t)ide analogue; ULN, upper limit of normal.

In the post hoc analysis of qHBsAg data collected at Centre 1, 263/457 (57.5%) people had complete qHBsAg data. Two stratifications of qHBsAg were explored: broad and granular. For the broad stratification of data from Centre 1, 167 (63.5%) had qHBsAg levels ≤ 3000 IU/mL and 96 (36.5%) had levels > 3000 IU/mL (Figure 1; Table 3). The proportion of males was higher in the qHBsAg ≤ 3000 IU/mL versus the > 3000 IU/mL group (53.9% [46.3–61.5] vs. 35.4% [25.8–45.0]). The mean (SD) age in the qHBsAg ≤ 3000 IU/mL stratum (49.6 [12.7] years; 95% CI: 47.7–51.5) was higher than in the > 3000 IU/mL stratum (43.5 [12.4] years; 95% CI: 41.0–46.0). People with qHBsAg ≤ 3000 IU/mL were more likely to be Asian (40.7% [33.3–48.2]) and less likely to be Black (13.2% [8.0–18.3]) than those with qHBsAg > 3000 IU/mL (Asian: 20.8% [12.7–29.0]; Black: 34.4% [24.9–43.9]). A greater proportion of people with qHBsAg ≤ 3000 IU/mL had undetectable HBV DNA versus those with qHBsAg > 3000 IU/mL (35.9% [28.7–43.2] vs. 17.7% [10.1–25.3]). There were proportionally more people with qHBsAg ≤ 3000 IU/mL having CKD than those with qHBsAg > 3000 IU/mL (8.4% [4.2–12.6] vs. 2.1 [0.3–7.3]), but 95% CIs overlapped.

**TABLE 3:** Characteristics of the study cohort by broad qHBsAg stratification (Centre 1).

	HBsAg level ≤ 3000 IU/mL (*n* = 167)			HBsAg level > 3000 IU/mL (*n* = 96)			HBsAg level missing (*n* = 194)		
	*n*	%	95% CI	*n*	%	95% CI	*n*	%	95% CI
Male	90	53.9	46.3–61.5	34	35.4	25.8–45.0	86	44.3	37.3–51.3
Age, years									
Mean (SD)	49.6 (12.7)			43.5 (12.4)			45.3 (14.5)		
Median (IQR)	49.0 (41.0–58.0)			44.0 (34.8–51.0)			44.0 (35.0–55.0)		
Age group									
< 25	4	2.4	0.7–6.0	5	5.2	1.7–11.7	10	5.2	2.1–8.3
25–49 years	92	55.1	47.5–62.6	64	66.7	57.2–76.1	116	59.8	52.9–66.7
50–64 years	47	28.1	21.3–35.0	21	21.9	13.6–30.1	48	24.7	18.7–30.8
65+ years	24	14.4	9.1–19.7	6	6.2	2.3–13.1	20	10.3	6.0–14.6
Ethnicity^a^									
White	48	28.7	21.9–35.6	25	26.0	17.3–34.8	48	24.7	18.7–30.8
Mixed	1	0.6	0–3.3	1	1.0	0–5.7	12	6.2	2.8–9.6
Asian	68	40.7	33.3–48.2	20	20.8	12.7–29.0	50	25.8	19.6–31.9
Black	22	13.2	8.0–18.3	33	34.4	24.9–43.9	52	26.8	20.6–33.0
Other/unknown	28	16.8	11.1–22.4	17	17.7	10.1–25.3	32	16.5	11.3–21.7
Co‐infections (HIV and/or HCV)	3	1.8	0.4–5.2	0			5	2.6	0.9–5.9
Hepatitis delta	3	1.8	0.4–5.2	0			2	1.0	0.1–3.7
Liver fibrosis	10	6.0	2.4–9.6	3	3.1	0.6–8.8	6	3.1	1.1–6.6
Compensated cirrhosis	11	6.6	2.8–10.4	3	3.1	0.6–8.9	8	4.1	1.8–8.0
Decompensated cirrhosis	0			0			0		
HCC	0			0			0		
CKD	14	8.4	4.2–12.6	2	2.1	0.3–7.3	10	5.2	2.0–8.3
qHBsAg, mean (SD)	762.5, 842.5			16,013.6, 24,899				—	
HBeAg status									
Positive	7	4.9	2.0–9.8	8	9.1	4.0–17.1	19	10.3	5.9–14.7
Negative	137	95.1	90.2–98.0	80	90.9	82.9–96.0	165	89.7	85.3–94.1
Missing	23	13.8	8.5–19.0	8	8.3	3.7–15.8	10	5.2	2.0–8.3
HBV DNA, IU/mL									
Undetectable	60	35.9	28.7–43.2	17	17.7	10.1–25.3	31	16.1	10.9–21.4
Detectable— < 2000	85	50.9	43.3–58.5	52	54.2	44.2–64.1	128	66.7	60.0–73.3
2000– < 20,000	19	11.4	6.6–16.2	20	20.8	12.7–29	22	11.5	7.0–16.0
≥ 20,000	3	1.8	0.4–5.2	7	7.3	3.0–14.4	11	5.7	2.4–9.0
Missing	0			0			2	1.0	0.1–3.7
ALT									
≤ ULN	155	94.5	89.8–97.5	82	85.4	78.4–92.5	166	88.8	84.2–93.3
1– < 2 × ULN	7	4.3	1.7–8.6	10	10.4	4.3–16.5	19	10.2	5.8–14.5
2– < 5 × ULN	2	1.2	0.1–4.3	1	1.0	0–5.7	2	1.1	0.1–3.8
≥ 5 × ULN	0			3	3.1	0.6–8.9	0		
Missing	3	1.8	0.4–5.2	0			7	3.6	1.5–7.3
Treatment									
NA monotherapy	95	56.9	49.4–64.4	48	50.0	40.0–60.0	57	29.4	23.0–35.8
NA combination	6	3.6	1.3–7.7	1	1	0–5.7	0		
Prior IFN use	0			0			0		
No treatment	66	39.5	32.1–46.9	47	49.0	39.0–59.0	137	70.6	64.2–77.0

*Note:* The table shows descriptive baseline data only; no formal hypothesis testing was conducted.

Abbreviations: ALT, alanine aminotransferase; CI, confidence interval; CKD, chronic kidney disease; HBeAg, hepatitis B e antigen; HBsAg, hepatitis B surface antigen; HBV, hepatitis B virus; HCC, hepatocellular carcinoma; HCV, hepatitis C virus; HIV, human immunodeficiency virus; IFN, interferon; IQR, interquartile range; NA, nucleos(t)ide analogue; qHBsAg, quantitative HBsAg; SD, standard deviation; ULN, upper limit of normal.

^a^ White = ‘White—British’, ‘White—Irish’, ‘White—any other White background’; Asian = ‘Asian or Asian British—Indian Asian or Asian British—Pakistani’, ‘Asian or Asian British—Bangladeshi’, ‘Asian or Asian British—any other Asian background’, ‘Chinese’; Black = ‘Black or Black British—Caribbean’, ‘Black or Black British—African’, ‘Black or Black British—any other Black background’; Mixed = ‘Mixed—White and Black Caribbean’, ‘Mixed—White and Black African’, ‘Mixed—White and Asian/Mixed—any other mixed background'; Other = ‘any other ethnic group’. Ethnicity was self‐reported by individuals and recorded using standard national codes in the UK.

**FIGURE 2:**
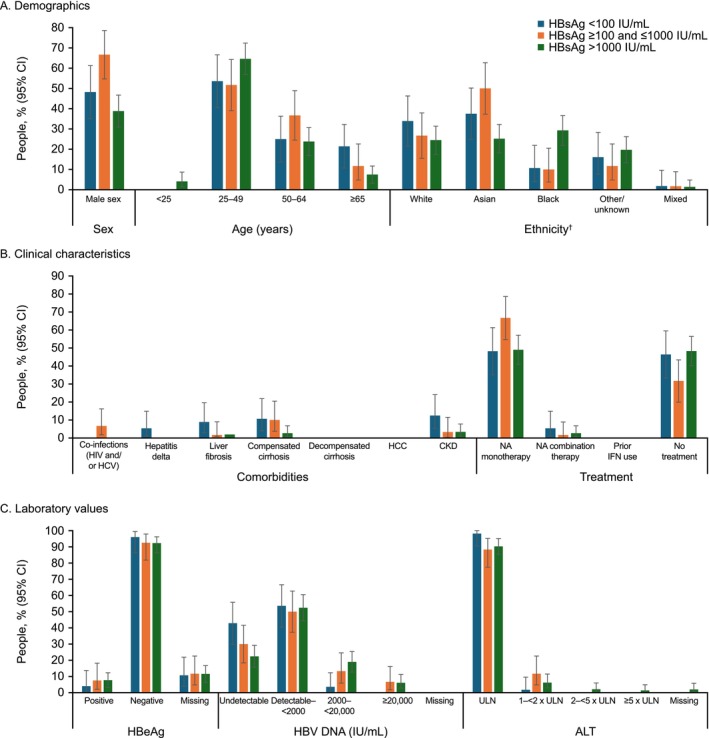
Characteristics of the study cohort by granular qHBsAg stratification: (A) demographics, (B) clinical characteristics and (C) laboratory values (Centre 1). White = ‘White—British’, ‘White—Irish’, ‘White—any other White background’; Asian = ‘Asian or Asian British—Indian Asian or Asian British—Pakistani’, ‘Asian or Asian British—Bangladeshi’, ‘Asian or Asian British—any other Asian background’, ‘Chinese’; Black = ‘Black or Black British—Caribbean’, ‘Black or Black British—African’, ‘Black or Black British—any other Black background’; Mixed = ‘Mixed—White and Black Caribbean’, ‘Mixed—White and Black African’, ‘Mixed—White and Asian/Mixed—any other mixed background'; Other = ‘any other ethnic group’. Ethnicity was self‐reported by individuals and recorded using standard national codes in the UK. ALT, alanine aminotransferase; CI, confidence interval; CKD, chronic kidney disease; HBeAg, hepatitis B e antigen; HBsAg, hepatitis B surface antigen; HBV, hepatitis B virus; HCC, hepatocellular carcinoma; HCV, hepatitis C virus; HIV, human immunodeficiency virus; IFN, interferon; NA, nucleos(t)ide analogue; ULN, upper limit of normal.

For the granular stratification of data from Centre 1, 56 (21.3%) people had qHBsAg levels < 100 IU/mL, 60 (22.8%) had levels ≥ 100–1000 IU/mL, and 147 (55.9%) had levels > 1000 IU/mL (Figure 2; Table 4). There were proportionally more males than females in the ≥ 100–1000 IU/mL stratum (66.7% [54.7–78.6] vs. 33.3% [21.4–45.3]). Conversely, the proportion of females was higher in the qHBsAg > 1000 IU/mL stratum (61.2% [53.3–69.1] vs. 38.8% [30.9–46.7]). There were no notable differences between sexes in the qHBsAg < 100 IU/mL stratum. Mean (SD) ages were higher in the qHBsAg < 100 IU/mL (51.9 [13.0] years; 95% CI: 48.5–55.3) and ≥ 100–1000 IU/mL (50.6 [12.5] years; 95% CI: 47.4–53.8) strata than in the > 1000 IU/mL (44.3 [12.2] years; 95% CI: 42.3–46.3) stratum. There was a greater proportion of people aged ≥ 65 years in the qHBsAg < 100 IU/mL stratum (21.4% [10.7–32.2]), versus the ≥ 100–1000 IU/mL (11.7% [4.8–22.6]) and > 1000 IU/mL (7.5% [3.2–11.7]) strata, although 95% CI values overlapped between strata. In the qHBsAg ≥ 100–1000 IU/mL stratum, there were proportionally more Asian people (50.0% [37.3–62.7]) and fewer Black people (10.0% [3.8–20.5]) than in the qHBsAg > 1000 IU/mL stratum (Asian: 25.2% [18.2–32.2]; Black: 29.3% [21.9–36.6]). In the qHBsAg < 100 IU/mL stratum, 37.5% (24.8–50.2) of people were Asian and 10.7% (4.0–21.9) were Black.

**TABLE 4:** Characteristics of the study cohort by granular qHBsAg stratification (Centre 1).

	HBsAg level < 100 IU/mL (*n* = 56)			HBsAg level ≥ 100– ≤ 1000 IU/mL (*n* = 60)			HBsAg level > 1000 IU/mL (*n* = 147)		
	*n*	%	95% CI	*n*	%	95% CI	*n*	%	95% CI
Male	27	48.2	35.1–61.3	40	66.7	54.7–78.6	57	38.8	30.9–46.7
Age, years									
Mean (SD)	51.9 (13.0)			50.6 (12.5)			44.3 (12.2)		
Median (IQR)	49.0 (43.8–62.3)			49.0 (41.8–60.0)			44.0 (35.0–52.0)		
Age group									
< 25 years	0			0			6	4.1	1.5–8.7
25–49 years	30	53.6	40.5–66.6	31	51.7	39.0–64.3	95	64.6	56.9–72.4
50–64 years	14	25.0	13.7–36.3	22	36.7	24.5–48.9	35	23.8	16.9–30.7
65+ years	12	21.4	10.7–32.2	7	11.7	4.8–22.6	11	7.5	3.2–11.7
Ethnicity^a^									
White	19	33.9	21.5–46.3	16	26.7	15.5–37.9	36	24.5	17.5–31.4
Mixed	1	1.8	0–9.6	1	1.7	0–8.9	2	1.4	0.2–4.8
Asian	21	37.5	24.8–50.2	30	50.0	37.3–62.7	37	25.2	18.2–32.2
Black	6	10.7	4.0–21.9	6	10.0	3.8–20.5	43	29.3	21.9–36.6
Other/unknown	9	16.1	7.6–28.3	7	11.7	4.8–22.6	29	19.7	13.3–26.2
Co‐infections (HIV and/or HCV)	0			4	6.7	1.8–16.2	0		
Liver fibrosis	5	8.9	3.0–19.6	1	1.7	0.8–9	3	2.0	0.4–5.8
Compensated cirrhosis	6	10.7	4.0–21.9	6	10.0	3.8–20.5	4	2.7	0.7–6.8
Decompensated cirrhosis	0			0			0		
HCC	0			0			0		
CKD	7	12.5	5.2–24.1	2	3.3	0.4–11.5	5	3.4	1.1–7.8
qHBsAg, mean (SD)	22.6, 29.8			504.3, 239.3			11,109.6, 21,191.7		
HBeAg status									
Positive	2	4.0	0.5–13.7	4	7.5	2.1–18.2	10	7.7	3.1–12.3
Negative	48	96.0	86.3–99.5	49	92.5	81.8–97.9	120	92.3	86.3–96.2
Missing	6	10.7	4–21.9	7	11.7	4.8–22.6	17	11.6	6.4–16.7
HBV DNA, IU/mL									
Undetectable	24	42.9	29.9–55.8	18	30.0	18.4–41.6	33	22.4	15.7–29.2
Detectable— < 2000	30	53.6	40.5–66.6	30	50.0	37.3–62.7	77	52.4	44.3–60.5
2000– < 20,000	2	3.6	0.4–12.3	8	13.3	5.9–24.6	28	19.0	12.7–25.4
≥ 20,000	0			4	6.7	1.8–16.2	9	6.1	2.8–11.3
Missing	0			0			0		
ALT									
≤ ULN	55	98.2	90.4–100	53	88.3	77.4–95.2	130	90.3	85.4–95.1
1– < 2 × ULN	1	1.8	0–9.6	7	11.7	4.8–22.6	9	6.2	2.9–11.5
2– < 5 × ULN	0			0			3	2.1	0.4–6.0
≥ 5 × ULN	0			0			2	1.4	0.2–4.9
Missing	0			0			3	2.0	0.4–5.8
Treatment									
NA monotherapy	27	48.2	35.1–61.3	40	66.7	54.7–78.6	72	49.0	40.9–57.1
NA combination	3	5.4	1.1–14.9	1	1.7	0–8.9	4	2.7	0.7–6.8
Prior IFN use	0			0			0		
No treatment	26	46.4	33.4–59.5	19	31.7	19.9–43.4	71	48.3	40.2–56.4

*Note:* The table shows descriptive baseline data only; no formal hypothesis testing was conducted.

Abbreviations: ALT, alanine aminotransferase; CI, confidence interval; CKD, chronic kidney disease; HBeAg, hepatitis B e antigen; HBsAg, hepatitis B surface antigen; HBV, hepatitis B virus; HCC, hepatocellular carcinoma; HCV, hepatitis C virus; HIV, human immunodeficiency virus; IFN, interferon; IQR, interquartile range; NA, nucleos(t)ide analogue; qHBsAg, quantitative HBsAg; SD, standard deviation; ULN, upper limit of normal.

^a^ White = ‘White—British’, ‘White—Irish’, ‘White—any other White background’; Asian = ‘Asian or Asian British—Indian Asian or Asian British—Pakistani’, ‘Asian or Asian British—Bangladeshi’, ‘Asian or Asian British—any other Asian background’, ‘Chinese’; Black = ‘Black or Black British—Caribbean’, ‘Black or Black British—African’, ‘Black or Black British—any other Black background’; Mixed = ‘Mixed—White and Black Caribbean’, ‘Mixed—White and Black African’, ‘Mixed—White and Asian/Mixed—any other mixed background'; Other = ‘any other ethnic group’. Ethnicity was self‐reported by individuals and recorded using standard national codes in the UK.

For laboratory values and clinical characteristics with granular stratification, the highest proportion of people with undetectable HBV DNA was in the qHBsAg < 100 IU/mL group (42.9% [29.9–55.8]), followed by the ≥ 100–1000 IU/mL group (30.0% [18.4–41.6]) and the > 1000 IU/mL group (22.4% [15.7–29.2]). People with lower qHBsAg levels were more likely to have CKD (12.5% [5.2–24.1] with < 100 IU/mL vs. 3.3% [0.4–11.5] with ≥ 100–1000 IU/mL and 3.4% [95% CI 1.1–7.8] with > 1000 IU/mL), although the 95% CI overlapped. There was increased NA monotherapy use in the qHBsAg ≥ 100–1000 IU/mL group (66.7% [54.7–78.6]) compared with the other qHBsAg groups (< 100 IU/mL: 48.2% [35.1–61.3]; > 1000 IU/mL: 49.0% [40.9–57.1]), although 95% CI values overlapped. Similarly, there were proportionally more untreated people with qHBsAg > 1000 IU/mL (48.3% [40.2–56.4]) and with qHBsAg < 100 IU/mL (46.4% [33.4–59.5]) compared with ≥ 100–1000 IU/mL (31.7% [19.9–43.4]), but again, 95% CI values overlapped.

Characteristics of the study cohort in Centre 2 stratified by semi‐quantitative HBsAg levels were broadly in line with the qHBsAg data from Centre 1, with people in the < 100 S/CO stratum being older, more likely to be Asian and have undetectable HBV DNA levels or CKD, and less likely to be Black than people in higher S/CO strata (Table S8).

## 3 Discussion

In line with recent studies exploring the relationship between qHBsAg levels and individual/disease characteristics [30, 31, 32, 33], the results of this large, ethnically diverse, real‐world study of people with chronic HBV infection reiterate the importance of assessing qHBsAg distribution in clinical settings. Quantification of HBsAg is not yet routine in clinical practice and was not incorporated in clinical guidelines at the time of this study [10, 21].

We first compared differences in individual characteristics between sites that collected and did not collect HBsAg levels. People with complete data were more often treated and were more likely to have undetectable HBV DNA; these findings suggest qHBsAg testing is more often undertaken to evaluate the effect of therapy and potentially to monitor those people considered to have a higher chance of HBsAg loss. Indeed, previous work has also shown that qHBsAg can be a useful biomarker to help healthcare providers identify individuals for whom functional cure is most likely to be achieved [34]. It can also be a useful diagnostic tool for predicting HBV reactivation [35].

Two of the four sites included in this study routinely undertook quantitative and semi‐quantitative HBsAg testing. The *post hoc* analysis of individuals with qHBsAg data showed that those with qHBsAg ≤ 3000 IU/mL represented the majority (63.5%) of those with available qHBsAg levels. Within the narrower stratification, people with lower qHBsAg levels (≤ 1000 IU/mL) constituted the minority group (44.1%). Compared with individuals with high qHBsAg levels (> 3000 IU/mL), those with lower levels were more likely to be older, of Asian ethnicity, and virologically suppressed. Small numbers within the narrower stratification made further comparisons challenging, but findings were consistent with respect to age (people with lower levels were more often older), ethnicity (people with lower levels were more often Asian), and, to a lesser extent, HBV DNA levels (people with lower qHBsAg levels tended to have lower viral loads). NA usage at baseline was more common in people with qHBsAg ≥ 100–1000 IU/mL compared with those with qHBsAg < 100 IU/mL and ≥ 1000 IU/mL.

Increased prevalence of cirrhosis was observed in individuals with low baseline qHBsAg levels (< 100 IU/mL) in this study, contrasting with a previous observational study in Japan where low baseline qHBsAg levels were significantly associated with a reduced risk of cirrhosis [36]. These results could potentially reflect more extensive use of this marker in the Centre 1 cohort for monitoring cirrhosis in treated individuals; however, the proportion of people with cirrhosis was 3.9% in both those with complete and missing qHBsAg or semi‐quantitative HBsAg data in Centres 1 and 2. In addition, the study population was ethnically diverse, thereby representing a broader spectrum of HBV genotypes and cohort settings than the Japanese study, which could explain the observed differences [36]. Furthermore, our results need to be interpreted with caution owing to the limited number of cirrhosis cases in this cohort and the unadjusted analytical approach, which does not control for potential confounding factors (e.g., age and HBeAg status). Such analyses were beyond the scope of this initial descriptive cross‐sectional study; future research leveraging longitudinal data to fully describe individual risk, and allying this with appropriate methods to minimize confounding factors, would inform further on this finding.

In a Korean study that examined the predictors of qHBsAg levels among untreated people with chronic HBV infection, age was reported to be inversely associated with qHBsAg levels in groups testing HBeAg‐positive and HBeAg‐negative, as well as in the entire sample (individuals testing HBeAg‐positive and HBeAg‐negative combined, *n* = 768) [37]. A positive association between age and the likelihood of HBsAg seroclearance was also demonstrated in two additional studies [38, 39]. The association between older age and the increased odds of HBsAg seroclearance is consistent with the findings of the present study, in which people in lower qHBsAg strata had a higher mean age.

This is the first study to describe the relationship between qHBsAg and ethnicity, with Asian people tending to have lower qHBsAg levels than people of other ethnicities, which could be important given that qHBsAg levels may be predictors of HBsAg and therapeutic response [40, 41]. However, no adjustment for confounding or covariates was made; further research would be required to establish any differences. Median qHBsAg levels in a Chinese population varied significantly [42], highlighting variability consistent with findings in a European cohort [35]. Studies examining predictors of HBsAg seroclearance among untreated people with chronic HBV infection in the USA and Canada, showed that non‐Asian ethnicity was independently associated with an increase in the odds of HBsAg seroclearance [38], and shorter time to HBsAg seroclearance [39]. These findings could be considered contradictory to ours if lower qHBsAg levels were *de facto* associated with an increased likelihood of HBsAg loss, but neither of the previous studies described qHBsAg levels by ethnicity. However, as mentioned above, no adjustment for confounding was performed in our study, so the observed relationship between lower qHBsAg and ethnicity may be due to other underlying factors. The discrepancy between our study and the US and Canadian studies could also be due to different racial make‐up within Asian and non‐Asian people in each setting. The wide categorizations used for ethnicity in this analysis preclude detailed investigation of any differences.

The differences in HBV DNA levels between higher and lower qHBsAg strata observed in this study are consistent with multiple published studies, which reported a positive correlation between qHBsAg levels and serum HBV DNA in various individual groups using unadjusted [43, 44, 45, 46, 47] and adjusted analyses [37, 48], suggesting that HBsAg levels are higher early in infection and diminish over time.

In this study, no differences in HBeAg positivity between qHBsAg strata were observed. Published literature on this is inconclusive [37, 43, 47, 49, 50]. Additionally, no differences were detected in ALT levels between qHBsAg strata. These findings are consistent with a study of 220 people with treatment‐naïve chronic HBV in Australia, which found no correlation between qHBsAg and ALT levels in the unadjusted analysis [45], while a Korean study reported a null association in the adjusted analysis of the combined HBeAg‐negative and HBeAg‐positive population, as well as within HBeAg strata [37].

The current study has several limitations, including issues commonly associated with EHR databases, such as limited data completeness and accuracy. Missing data, miscoded information, or inaccuracies in recorded clinical diagnoses may introduce bias and result in misclassification errors. Only one site provided qHBsAg level data, with another site contributing semi‐quantitative HBsAg data, which limits internal validity. However, this limitation is outweighed by the external validity gained from the generalisability of findings to routine clinical practice, compared with data from clinical trials, which may be more accurate and complete but less reflective of real‐world populations. Additionally, the study's cross‐sectional design and reliance on a single measurement of qHBsAg per person precluded analysis of the longitudinal dynamics of qHBsAg levels over time. Indeed, tracking of temporal intra/interindividual variability of ALT levels could have contributed to better dissecting the association between ALT levels and qHBsAg strata. Further research is needed to identify predictors of qHBsAg levels and to elucidate their kinetics. This study was descriptive; no adjustment for confounding or covariates was performed, so the effects of some variables on others could not be excluded; multivariable analyses would be required to further ascertain patterns of characteristics in people with chronic HBV infection. Further, the sample size did not allow reaching enough power for performing more complex analyses and the number of people in some strata groups was low, rendering wide 95% CIs and thus limiting the conclusions that can be drawn.

The generalisability of findings from this study to the broader UK healthcare setting and, by extension, to the rest of Europe, presents potential limitations. The combined data from the contributing collaborators may not fully capture the clinical and biochemical characteristics of people with chronic HBV infection across the UK. Furthermore, the publicly funded healthcare system in the UK may be more comparable to some European countries (e.g., Spain and Italy), but differs significantly from countries with predominantly insurance‐based healthcare systems (e.g., France and Germany). This discrepancy limits the generalisability of findings to Europe as a whole.

In summary, people with lower HBsAg levels (≤ 3000 IU/mL) were more likely to be older, of Asian ethnicity, and virologically suppressed than those with higher HBsAg levels (> 3000 IU/mL). Despite limitations, insights from this study demonstrate the value of routine qHBsAg testing in real‐world clinical practice and could help identify potential populations who may benefit from emerging anti‐HBV therapies and therefore be more likely to achieve a functional cure.

## Author Contributions

M.D., T.W., P.C.M., J.D., K.W., G.R., G.S.C., S.I.K., E.N., I.A.G. and E.B. contributed to the conception or design of the study; T.W., J.D., K.W., G.R., S.L., H.S., K.A.V., T.N., G.S.C., B.G., D.P., E.M., S.I.K., C.D., F.B., E.N., L.E. and E.B. contributed to data acquisition; T.W., D.C. and I.A.G. contributed to data analysis; M.D., D.C., P.C.M., G.S.C., S.I.K., E.N., I.A.G. and E.B. contributed to data interpretation. All authors reviewed and approved the final manuscript.

## Conflicts of Interest

M.D., I.A.G. and D.C. are employees of GSK and hold financial equities in GSK. E.B. holds patents in HBV vaccines and has received funding for research from GSK and Barinthus in the field of HBV. P.C.M. received GSK funding support for a doctoral research fellow in her group between 2019 and 2023. J.D., K.W., G.R., S.L., H.S., K.A.V., T.N., G.S.C., B.G., D.P., E.M., S.I.K., C.D., F.B., L.E., E.N. and T.W. have no conflicts of interest to declare.

## Supporting information


**Data S1:** jvh70101‐sup‐0001‐DataS1.docx.

## Data Availability

For requests for access to anonymised subject‐level data, please contact Myriam Drysdale.
