# Supplementary material for: Characterisation of People Living With Chronic Hepatitis B Virus Infection in England and Stratification by HBsAg Levels: A Cross‐Sectional Study
Source: J Viral Hepat. 2025 Nov 20;32(12):e70101. doi: 10.1111/jvh.70101 (PMC12631719; doi:10.1111/jvh.70101)
Supplement: Supplementary file 1 — Data S1: jvh70101‐sup‐0001‐DataS1.docx. [file JVH-32-0-s001.docx]

# Supplementary Material

## Supplementary Figure 1: Study cohort


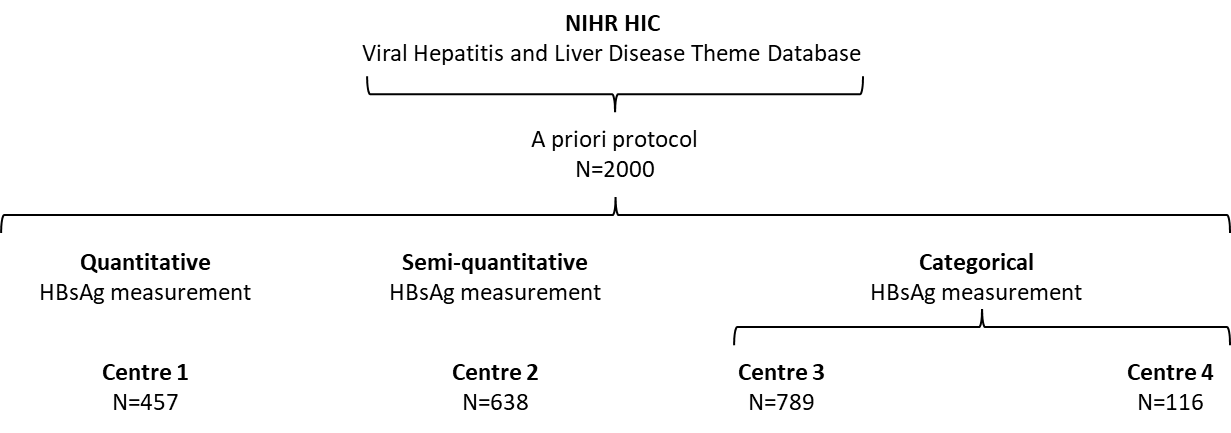


The initial NHIR HIC collaboration included four sites, selected based on their data collation status at the time of study initiation.
HBsAg, hepatitis B surface antigen; NIHR HIC, National Institute for Health and Care Research Health Informatics Collaborative.

## Supplementary Figure 2: Study design


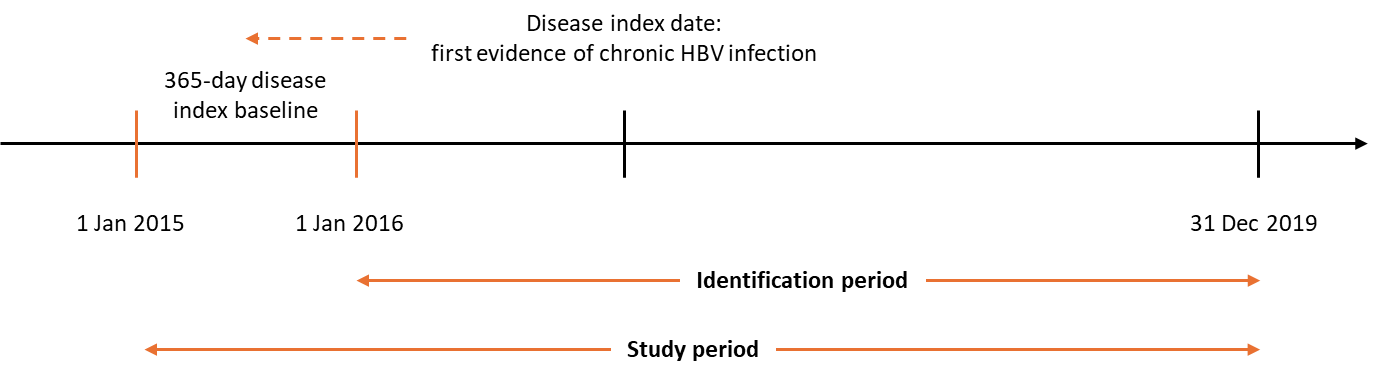


HBV, hepatitis B virus.

## Supplementary Table 1: Eligibility criteria for the study cohort

| **Inclusion criteria** | **Exclusion criteria** |
| --- | --- |
| Two positive HBsAg tests^†^, at least 6 months apart, with at least one occurring during the identification period. The second positive test is the disease index date. | People with acute HBV, defined as:   - Positive IgM to hepatitis B core antigen (unless >6 months from a positive HBsAg result). - Conversion from positive to negative HBsAg within 6 months (unless two positive HBsAg results occurred ≥6 months before the negative result). |
| Positive HBsAg plus positive HBV DNA at least 6 months apart, with at least one occurring during the identification period. The second positive test is the disease index date. | People with a single HBsAg test that cannot conclusively establish chronic HBV. |
| A single positive HBsAg during the identification period, forming the disease index date, only if acute HBV is actively excluded. |  |

†Note that the HIC definition includes individuals with only one positive HBsAg test; however, for this present study, the definition was two positive tests.
HBsAg, hepatitis B surface antigen; HBV, hepatitis B virus; HIC, Health Informatics Collaborative; IgM, immunoglobulin M.

## Supplementary Table 2: Cohort selection for this study

|  | Number of individuals who meet the pre-defined eligibility criteria | Number of individuals included to reach the target sample size as per study protocol |
| --- | --- | --- |
| Centre 1 | 457 | 457 |
| Centre 2 | 638 | 638 |
| Centre 3 | 116 | 116 |
| Centre 4 | 1668 | 789 randomly selected |
| Total | 2879 | 2000 |

## Supplementary Table 3: Outcome variables

| **Type of variable** | **Variable** | **Categories** |
| --- | --- | --- |
| **Demographics** | Sex | Male, Female, Unknown |
|  | Ethnicity^†^ | White, Mixed, Asian, Black, other/unknown |
| **Infection characteristics (within 365 days of baseline unless stated)** | Co-infection | HCV alone, HIV alone, both HIV and HCV, None (Note: any history of HIV) |
|  | Hepatitis delta co-infection | Yes, No |
| **Clinical (within 365 days of baseline unless stated)** | Liver fibrosis^‡^ | Yes, No |
|  | Cirrhosis^‡^ (any history) | No, Compensated, Decompensated^§^ |
|  | HCC^§^ (any history) | Yes, No |
|  | Liver transplant (any history) | Yes, No |
|  | CKD^¶^ | Yes, No |
| **Laboratory measurements (last observation)** | qHBsAg level (IU/mL) | <100, ≥100–≤1000, >1000, Missing |
|  | qHBsAg (continuous variable) | N observations, Min, Max, Mean (SD), Median [IQR] |
|  | HBeAg status | Positive, Negative, Missing |
|  | HBV DNA | Undetectable, Detectable (<2000, 2000–<20,000, ≥20,000), Missing |
|  | ALT ULN | ≤ ULN, 1–<2 × ULN, 2–<5 × ULN, ≥5 × ULN, Missing |
| **Treatment at disease index** | Treatment status | No antiviral treatment, IFN monotherapy (Type: IFN alpha/PEG-IFN), NA monotherapy (Type: Lamivudine/Adefovir/Telbivudine/Entecavir/Tenofovir; Prior IFN use),  Drug combinations (IFN+NA, Combinations of NAs), Missing |

†White = ^’^White – British’, ‘White – Irish’, ‘White - any other White background’; Asian = ‘Asian or Asian British – Indian Asian or Asian British – Pakistani’, ‘Asian or Asian British – Bangladeshi’, ‘Asian or Asian British – Any other Asian background’, ‘Chinese’; Black = ‘Black or Black British – Caribbean’, ‘Black or Black British – African’, ‘Black or Black British – any other Black background’; Mixed = ‘Mixed – White and Black Caribbean’, ‘Mixed – White and Black African’, ’Mixed – White and Asian/Mixed – Any other mixed background’; Other = ‘Any other ethnic group’. Ethnicity was self-reported by individuals and recorded using standard national codes in the UK.

‡Fibrosis was defined as a biopsy Ishak score of ≥1 and <6, an elastography liver stiffness of ≥6 and <11 kPa, or if biopsy and elastography are not available, APRI score >1.5 and ≤2 or a FIB-4 score >3.25 and ≤3.6; cirrhosis was defined as a biopsy Ishak score of 6, an elastography liver stiffness of ≥11 kPa, or if biopsy and elastography are not available, APRI score >2 or a FIB-4 score >3.6.

§Decompensation and HCC defined from biopsy reports or imaging if biopsy not available.

¶Two measurements at least 90 days apart confirming CKD were required.

ALT, alanine aminotransferase; APRI, AST to platelet ratio index; CKD, chronic kidney disease; FIB-4, fibrosis-4; HBV, hepatitis B virus; HBeAg, hepatitis B e antigen; HBsAg, hepatitis B surface antigen; HCC, hepatocellular carcinoma; HCV, hepatitis C virus; HIV, human immunodeficiency virus; IFN, interferon; IQR, interquartile range; NA, nucleos(t)ide analogue; qHBsAg, quantitative HBsAg; ULN, upper limit of normal.

## Supplementary Table 4: Characteristics of the study cohort by liver disease status

| **Variables** | **No liver disease (n=1809)** | | **Any evidence of liver disease (n=191)** | |
| --- | --- | --- | --- | --- |
|  | **n (%)** | **95% CI** | **n (%)** | **95% CI** |
| **Male sex** | 948 (52.4) | 50.1–54.7 | 131 (68.6) | 62.0–75.2 |
| **Age, years** |  |  |  |  |
| Mean (SD) | 44.1 (12.2) | | 52.7 (15.1) | |
| Median (IQR) | 43.0 (35.0–51.0) | | 55.0 (42.0–64.0) | |
| **Age group, years** |  |  |  |  |
| <25 | 42 (2.3) | 1.6–3.0 | 6 (3.1) | 1.1–6.7 |
| 25–49 | 1244 (68.8) | 66.6–70.9 | 77 (40.3) | 33.4–47.3 |
| 50–64 | 402 (22.2) | 20.3–24.1 | 65 (34.0) | 27.3–40.8 |
| ≥65 | 121 (6.7) | 5.5–7.8 | 43 (22.5) | 16.6–28.4 |
| **Ethnicity^†^** |  |  |  |  |
| White | 445 (24.6) | 22.6–26.6 | 50 (26.2) | 19.9–32.4 |
| Mixed | 55 (3.0) | 2.2–3.8 | 7 (3.7) | 1.5–7.4 |
| Asian | 453 (25.0) | 23.0–27.0 | 47 (24.6) | 18.5–30.7 |
| Black | 348 (19.2) | 17.4–21.1 | 35 (18.3) | 12.8–23.8 |
| Other/unknown | 508 (28.1) | 26.0–30.2 | 52 (27.2) | 20.9–33.5 |
| **Co-infections (HIV and/or HCV)** | 28 (1.5) | 0.9–2.1 | 10 (5.2) | 2.5–9.4 |
| **Hepatitis delta** | 4 (0.2) | 0.1–0.6 | 2 (1.0) | 0.1–3.7 |
| **Liver fibrosis** | 0 | – | 89 (46.6) | 39.5–53.7 |
| **Cirrhosis (any history)** |  |  |  |  |
| Compensated | 0 | – | 137 (71.7) | 65.3–78.1 |
| Decompensated | 0 | – | 18 (9.4) | 5.3–13.6 |
| **HCC (any history)** | 0 | – | 12 (6.3) | 2.8–9.7 |
| **CKD** | 73 (4.0) | 3.1–4.9 | 31 (16.2) | 11.0–21.5 |
| **qHBsAg** | **(n=246)** | | **(n=17)** | |
| Mean (SD) | 6412.6 (17,127.3) | | 5126.0 (9003.6) | |
| Median (IQR) | 1638.8 (250–5859.6) | | 509.4 (80.1–1383.4) | |
| **qHBsAg broad stratification** |  |  |  |  |
| ≤3000 IU/mL | 154 (62.6) | 56.6–68.6 | 13 (76.5) | 50.1–93.2 |
| >3000 IU/mL | 92 (37.4) | 31.4–43.4 | 4 (23.5) | 6.8–49.9 |
| Missing (Centre 1) | 183 (42.7) | 38.0–47.3 | 11 (39.3 ) | 21.2–57.4 |
| Missing (all centres) | 1563 (86.4) | 84.8–88 | 174 (91.1) | 87.1–95.1 |
| **qHBsAg granular stratification** |  |  |  |  |
| <100 IU/mL | 50 (20.3) | 15.3–25.4 | 6 (35.3) | 14.2–61.7 |
| ≥100 and ≤1000 IU/mL | 54 (22.0) | 16.8–27.1 | 6 (35.3) | 14.2–61.7 |
| >1000 IU/mL | 142 (57.7) | 51.6–63.9 | 5 (29.4) | 10.3–56.0 |
| Missing (Centre 1) | 183 (42.7) | 38.0–47.3 | 11 (39.3 ) | 21.2–57.4 |
| Missing (all centres) | 1563 (86.4) | 84.8–88 | 174 (91.1) | 87.1–95.1 |
| **HBeAg status** |  |  |  |  |
| Positive | 112 (8.7) | 7.2–10.3 | 21 (16.8) | 10.2–23.4 |
| Negative | 1174 (91.3) | 89.7–92.8 | 104 (83.2) | 76.6–89.8 |
| Missing | 523 (28.9) | 26.8–31.0 | 66 (34.6) | 27.8–41.3 |
| **HBV DNA, IU/mL** |  |  |  |  |
| Undetectable | 199 (11.3) | 9.9–12.8 | 31 (16.6) | 11.2–21.9 |
| Detectable–<2000 | 1318 (75.1) | 73.1–77.2 | 137 (73.3) | 66.9–79.6 |
| 2000–<20,000 | 172 (9.8) | 8.4–11.2 | 10 (5.3) | 2.1–8.6 |
| ≥20,000 | 65 (3.7) | 2.8–4.6 | 9 (4.8) | 2.2–8.9 |
| Missing | 55 (3.0) | 2.2–3.8 | 4 (2.1) | 0.6–5.3 |
| **ALT** |  |  |  |  |
| ≤ULN | 1526 (85.8) | 84.2–87.4 | 139 (72.8) | 66.5–79.1 |
| 1–<2 × ULN | 208 (11.7) | 10.2–13.2 | 31 (16.2) | 11–21.5 |
| 2–<5 × ULN | 39 (2.2) | 1.5–2.9 | 16 (8.4) | 4.4–12.3 |
| ≥5 × ULN | 5 (0.3) | 0.1–0.7 | 5 (2.6) | 0.9–6.0 |
| Missing | 31 (1.7) | 1.1–2.3 | 0 | – |
| **Treatment** |  |  |  |  |
| NA monotherapy | 368 (20.3) | 18.5–22.2 | 67 (35.1) | 28.3–41.8 |
| NA combination therapy | 17 (0.9) | 0.5–1.4 | 10 (5.2) | 2.1–8.4 |
| Prior IFN use | 2 (0.1) | 0–0.4 | 0 | – |
| No treatment | 1424 (78.7) | 76.8–80.6 | 114 (59.7) | 52.7–66.6 |

The table shows descriptive baseline data only; no formal hypothesis testing was conducted.

†White = ^’^White – British’, ‘White – Irish’, ‘White - any other White background’; Asian = ‘Asian or Asian British – Indian Asian or Asian British – Pakistani’, ‘Asian or Asian British – Bangladeshi’, ‘Asian or Asian British – Any other Asian background’, ‘Chinese’; Black = ‘Black or Black British – Caribbean’, ‘Black or Black British – African’, ‘Black or Black British – any other Black background’; Mixed = ‘Mixed – White and Black Caribbean’, ‘Mixed – White and Black African’, ’Mixed – White and Asian/Mixed – Any other mixed background’; Other = ‘Any other ethnic group’. Ethnicity was self-reported by individuals and recorded using standard national codes in the UK.
ALT, alanine aminotransferase; CI, confidence interval; CKD, chronic kidney disease; HBeAg, hepatitis B e antigen; HBsAg, hepatitis B surface antigen; HBV, hepatitis B virus; HCC, hepatocellular carcinoma; HCV, hepatitis C virus; HIV, human immunodeficiency virus; IFN, interferon; IQR, interquartile range; NA, nucleos(t)ide analogue; qHBsAg, quantitative HBsAg; SD, standard deviation; ULN, upper limit of normal.

## Supplementary Table 5: Characteristics of the study cohort by treatment status

| **Variables** | **No treatment (n=1538)** | | **NA monotherapy/combination therapy (n=462)** | |
| --- | --- | --- | --- | --- |
|  | **n (%)** | **95% CI** | **n (%)** | **95% CI** |
| **Male sex** | 785 (51.0) | 48.5–53.5 | 294 (63.6) | 59.2–68.0 |
| **Age, years** |  |  |  |  |
| Mean (SD) | 43.8 (12.4) |  | 48.7 (13.1) |  |
| Median (IQR) | 42.0 (35.0–51.0) |  | 48.0 (39.0–58.0) |  |
| **Age group, years** |  |  |  |  |
| <25 | 40 (2.6) | 1.8–3.4 | 8 (1.7) | 0.8–3.4 |
| 25–49 | 1070 (69.6) | 67.3–71.9 | 251 (54.3) | 49.8–58.9 |
| 50–64 | 324 (21.1) | 19.0–23.1 | 143 (31.0) | 26.7–35.2 |
| ≥65 | 104 (6.8) | 5.5–8.0 | 60 (13.0) | 9.9–16.1 |
| **Ethnicity^†^** |  |  |  |  |
| White | 375 (24.4) | 22.2–26.5 | 120 (26.0) | 22.0–30.0 |
| Mixed | 46 (3.0) | 2.1–3.8 | 16 (3.5) | 1.8–5.1 |
| Asian | 348 (22.6) | 20.5–24.7 | 152 (32.9) | 28.6–37.2 |
| Black | 308 (20.0) | 18.0–22.0 | 75 (16.2) | 12.9–19.6 |
| Other/unknown | 461 (30.0) | 27.7–32.3 | 99 (21.4) | 17.7–25.2 |
| **Co-infections (HIV and/or HCV)** | 28 (1.8) | 1.1–2.5 | 10 (2.2) | 0.8–3.5 |
| **Hepatitis delta** | 4 (0.3) | 0.1–0.7 | 2 (0.4) | 0.1–1.6 |
| **Liver fibrosis** | 55 (3.6) | 2.7–4.5 | 34 (7.4) | 5.0–9.8 |
| **Cirrhosis (any history)** |  |  |  |  |
| Compensated | 91 (5.9) | 4.7–7.1 | 46 (10.0) | 7.3–12.7 |
| Decompensated | 4 (0.3) | 0.1–0.7 | 14 (3.0) | 1.5–4.6 |
| **HCC (any history)** | 2 (0.1) | 0–0.5 | 10 (2.2) | 0.8–3.5 |
| **CKD** | 57 (3.7) | 2.8–4.7 | 47 (10.2) | 7.4–12.9 |
| **qHBsAg** | **(n=116)** | | **(n=147)** | |
| Mean (SD) | 7256.5 (13,706.6) | | 5597.9 (18,764.8) | |
| Median (IQR) | 2115.1 (250–8926.3) | | 1108.8 (202.5–4191.7) | |
| **qHBsAg broad stratification** |  |  |  |  |
| ≤3000 IU/mL | 69 (59.5) | 50.5–68.4 | 98 (66.7) | 59.0–74.3 |
| >3000 IU/mL | 47 (40.5) | 31.6–49.5 | 49 (33.3) | 25.7–41.0 |
| Missing (Centre 1) | 137 (54.2) | 48.0–60.3 | 57 (27.9) | 21.8–34.1 |
| Missing (all centres) | 1422 (92.5) | 91.1–93.8 | 315 (68.2) | 63.9–72.4 |
| **qHBsAg granular stratification** |  |  |  |  |
| <100 IU/mL | 26 (22.4) | 14.8–30.0 | 30 (20.4) | 13.9–26.9 |
| ≥100 and ≤1000 IU/mL | 19 (16.4) | 9.6–23.1 | 41 (27.9) | 20.6–35.1 |
| >1000 IU/mL | 71 (61.2) | 52.3–70.1 | 76 (51.7) | 43.6–59.8 |
| Missing (Centre 1) | 137 (54.2) | 48.0–60.3 | 57 (27.9) | 21.8–34.1 |
| Missing (all centres) | 1422 (92.5) | 91.1–93.8 | 315 (68.2) | 63.9–72.4 |
| **HBeAg status** |  |  |  |  |
| Positive | 75 (7.2) | 5.7–8.8 | 58 (15.5) | 11.8–19.2 |
| Negative | 962 (92.8) | 91.2–94.3 | 316 (84.5) | 80.8–88.2 |
| Missing | 501 (32.6) | 30.2–34.9 | 88 (19.0) | 15.5–22.6 |
| **HBV DNA, IU/mL** |  |  |  |  |
| Undetectable | 48 (3.2) | 2.3–4.1 | 182 (39.7) | 35.3–44.2 |
| Detectable–<2000 | 1209 (81.5) | 79.5–83.5 | 246 (53.7) | 49.1–58.3 |
| 2000–<20,000 | 158 (10.7) | 9.1–12.2 | 24 (5.2) | 3.2–7.3 |
| ≥20,000 | 68 (4.6) | 3.5–5.6 | 6 (1.3) | 0.5–2.8 |
| Missing | 55 (3.6) | 2.6–4.5 | 4 (0.9) | 0.2–2.2 |
| **ALT** |  |  |  |  |
| ≤ULN | 1270 (84.2) | 82.4–86.1 | 395 (85.7) | 82.5–88.9 |
| 1–<2 × ULN | 187 (12.4) | 10.7–14.1 | 52 (11.3) | 8.4–14.2 |
| 2–<5 × ULN | 44 (2.9) | 2.1–3.8 | 11 (2.4) | 1.0–3.8 |
| ≥5 × ULN | 7 (0.5) | 0.2–1.0 | 3 (0.7) | 0.1–1.9 |
| Missing | 30 (2.0) | 1.3–2.6 | 1 (0.2) | 0–1.2 |

The table shows descriptive baseline data only; no formal hypothesis testing was conducted.

†White = ^’^White – British’, ‘White – Irish’, ‘White - any other White background’; Asian = ‘Asian or Asian British – Indian Asian or Asian British – Pakistani’, ‘Asian or Asian British – Bangladeshi’, ‘Asian or Asian British – Any other Asian background’, ‘Chinese’; Black = ‘Black or Black British – Caribbean’, ‘Black or Black British – African’, ‘Black or Black British – any other Black background’; Mixed = ‘Mixed – White and Black Caribbean’, ‘Mixed – White and Black African’, ’Mixed – White and Asian/Mixed – Any other mixed background’; Other = ‘Any other ethnic group’. Ethnicity was self-reported by individuals and recorded using standard national codes in the UK.
ALT, alanine aminotransferase; CI, confidence interval; CKD, chronic kidney disease; HBeAg, hepatitis B e antigen; HBsAg, hepatitis B surface antigen; HBV, hepatitis B virus; HCC, hepatocellular carcinoma; HCV, hepatitis C virus; HIV, human immunodeficiency virus; IQR, interquartile range; NA, nucleos(t)ide analogue; qHBsAg, quantitative HBsAg; SD, standard deviation; ULN, upper limit of normal.

## Supplementary Table 6: Characteristics of the study cohort by HBeAg status

| **Variables** | **HBeAg-positive (n=133)** | | **HBeAg-negative (n=1278)** | | **HBeAg missing (n=589)** | |
| --- | --- | --- | --- | --- | --- | --- |
|  | **n (%)** | **95% CI** | **n (%)** | **95% CI** | **n (%)** | **95% CI** |
| **Male sex** | 79 (59.4) | 51.1–67.7 | 690 (54.0) | 51.3–56.7 | 310 (52.6) | 48.6–56.7 |
| **Age** |  |  |  |  |  |  |
| Mean (SD) | 39.6 (13.8) |  | 44.7 (12.4) |  | 46.6 (13.1) |  |
| Median (IQR) | 36.0 (29.0, 49.0) |  | 43.0 (36.0, 52.0) |  | 45.0 (37.0, 55.0) |  |
| **Age group** |  |  |  |  |  |  |
| <25 | 13 (9.8) | 4.7–14.9 | 20 (1.6) | 0.9–2.3 | 15 (2.5) | 1.2–3.8 |
| 25–49 | 89 (66.9) | 58.9–74.9 | 866 (67.8) | 65.2–70.3 | 366 (62.1) | 58.2–66.1 |
| 50–64 | 22 (16.5) | 10.2–22.9 | 295 (23.1) | 20.8–25.4 | 150 (25.5) | 21.9–29.0 |
| ≥65 | 9 (6.8) | 3.1–12.5 | 97 (7.6) | 6.1–9.0 | 58 (9.8) | 7.4–12.3 |
| **Ethnicity^†^** |  |  |  |  |  |  |
| White | 26 (19.5) | 12.8–26.3 | 318 (24.9) | 22.5–27.3 | 151 (25.6) | 22.1–29.2 |
| Mixed | 6 (4.5) | 1.7–9.6 | 38 (3.0) | 2.0–3.9 | 18 (3.1) | 1.7–4.4 |
| Asian | 51 (38.3) | 30.1–46.6 | 311 (24.3) | 22.0–26.7 | 138 (23.4) | 20.0–26.9 |
| Black | 12 (9.0) | 4.2–13.9 | 259 (20.3) | 18.1–22.5 | 112 (19.0) | 15.8–22.2 |
| Other/unknown | 38 (28.6) | 20.9–36.2 | 352 (27.5) | 25.1–30.0 | 170 (28.9) | 25.2–32.5 |
| **Co-infections (HIV and/or HCV)** | 6 (4.5) | 1.7–9.6 | 13 (1.0) | 0.5–1.5 | 19 (3.2) | 1.8–4.6 |
| **Hepatitis delta** | 0 |  | 4 (0.3) | 0.1–0.8 | 2 (0.3) | 0–1.2 |
| **Liver fibrosis** | 15 (11.3) | 5.9–16.7 | 53 (4.1) | 3.0–5.2 | 21 (3.6) | 2.1–5.1 |
| **Cirrhosis (any history)** |  |  |  |  |  |  |
| Compensated | 13 (9.8) | 4.7–14.9 | 72 (5.6) | 4.3–6.9 | 52 (8.8) | 6.5–11.1 |
| Decompensated | 2 (1.5) | 0.2–5.3 | 11 (0.9) | 0.4–1.4 | 5 (0.8) | 0.3–2.0 |
| **HCC (any history)** | 1 (0.8) | 0–4.1 | 6 (0.5) | 0.2–1.0 | 5 (0.8) | 0.3–2.0 |
| **CKD** | 11 (8.3) | 3.6–13 | 64 (5.0) | 3.8–6.2 | 29 (4.9) | 3.2–6.7 |
| **qHBsAg** | **(n=15)** |  | **(n=217)** |  | **(n=31)** |  |
| Mean (SD) | 33,218.3 (57,489.5) |  | 4795.5 (7796.9) |  | 4056.0 (8708.0) |  |
| Median (IQR) | 5394.2 (605.3–30,969.5) |  | 1356.5 (181.7–5329.6) |  | 1511.3 (223.1–3064.9) |  |
| **qHBsAg broad stratification** |  |  |  |  |  |  |
| ≤3000 IU/mL | 7 (46.7) | 21.3–73.4 | 137 (63.1) | 56.7–69.6 | 23 (74.2) | 55.4–88.1 |
| >3000 IU/mL | 8 (53.3) | 26.6–78.7 | 80 (36.9) | 30.4–43.3 | 8 (25.8) | 11.9–44.6 |
| Missing (Centre 1) | 19 (55.9) | 39.2–72.6 | 165 (43.2) | 38.2–48.2 | 10 (24.4) | 11.2–37.5 |
| Missing (all centres) | 118 (88.7) | 83.3–94.1 | 1061 (83.0)) | 81.0–85.1 | 558 (94.7) | 92.9–96.5 |
| **qHBsAg granular stratification** |  |  |  |  |  |  |
| <100 IU/mL | 1 (6.7) | 0.2–31.9– | 48 (22.1) | 16.6–27.6 | 7 (22.6) | 9.6–41.1 |
| ≥100 and ≤1000 IU/mL | 4 (26.7) | 7.8–55.1 | 49 (22.6) | 17.0–28.1 | 7 (22.6) | 9.6–41.1 |
| >1000 IU/mL | 10 (66.7) | 38.4–88.2 | 120 (55.3) | 48.7–61.9 | 17 (54.8) | 37.3–72.4 |
| Missing (Centre 1) | 19 (55.9) | 39.2–72.6 | 165 (43.2) | 38.2–48.2 | 10 (24.4 ) | 11.2–37.5 |
| Missing (all centres) | 118 (88.7) | 83.3–94.1 | 1061 (83.0)) | 81.0–85.1 | 558 (94.7) | 92.9–96.5 |
| **HBV DNA, IU/mL** |  |  |  |  |  |  |
| Undetectable | 11 (8.4) | 3.6–13.1 | 174 (13.9) | 12.0–15.8 | 45 (8.0) | 5.8–10.3 |
| Detectable–<2000 IU/mL | 80 (61.1) | 52.7–69.4 | 926 (74.1) | 71.7–76.5 | 449 (80.2) | 76.9–83.5 |
| 2000–<20,000 IU/mL | 11 (8.4) | 3.6–13.1 | 118 (9.4) | 7.8–11.1 | 53 (9.5) | 7.0–11.9 |
| ≥20,000 IU/mL | 29 (22.1) | 15.0–29.2 | 32 (2.6) | 1.7–3.4 | 13 (2.3) | 1.1–3.6 |
| Missing | 2 (1.5) | 0.2–5.3 | 28 (2.2) | 1.4–3.0 | 29 (4.9) | 3.2–6.7 |
| **ALT** |  |  |  |  |  |  |
| ≤ULN | 92 (69.2) | 61.3–77.0 | 1077 (85.5) | 83.6–87.5 | 496 (86.0) | 83.1–88.8 |
| 1–<2 × ULN | 29 (21.8) | 14.8–28.8 | 148 (11.8) | 10.0–13.5 | 62 (10.7) | 8.2–13.3 |
| 2–<5 × ULN | 10 (7.5) | 3.0–12.0 | 28 (2.2) | 1.4–3.0 | 17 (2.9) | 1.6–4.3 |
| ≥5 × ULN | 2 (1.5) | 0.2–5.3 | 6 (0.5) | 0.2–1.0 | 2 (0.3) | 0–1.2 |
| Missing | 0 |  | 19 (1.5) | 0.8–2.2 | 12 (2.0) | 0.9–3.2 |
| **Treatment** |  |  |  |  |  |  |
| NA monotherapy | 51 (38.3) | 30.1–46.6 | 305 (23.9) | 21.5–26.2 | 79 (13.4) | 10.7–16.2 |
| NA combination therapy | 7 (5.3) | 2.1–10.5 | 11 (0.9) | 0.4–1.4 | 9 (1.5) | 0.70–2.9 |
| Prior IFN use | 0 |  | 2 (0.2) | 0–0.6 | 0 |  |
| No treatment | 75 (56.4) | 48–64.8 | 962 (75.3) | 72.9–77.6 | 501 (85.1) | 82.2–87.9 |

The table shows descriptive baseline data only; no formal hypothesis testing was conducted.

†White = ^’^White – British’, ‘White – Irish’, ‘White - any other White background’; Asian = ‘Asian or Asian British – Indian Asian or Asian British – Pakistani’, ‘Asian or Asian British – Bangladeshi’, ‘Asian or Asian British – Any other Asian background’, ‘Chinese’; Black = ‘Black or Black British – Caribbean’, ‘Black or Black British – African’, ‘Black or Black British – any other Black background’; Mixed = ‘Mixed – White and Black Caribbean’, ‘Mixed – White and Black African’, ’Mixed – White and Asian/Mixed – Any other mixed background’; Other = ‘Any other ethnic group’. Ethnicity was self-reported by individuals and recorded using standard national codes in the UK.
ALT, alanine aminotransferase; HBeAg, hepatitis B e antigen; CI, confidence interval; CKD, chronic kidney disease; HBeAg, hepatitis B e antigen; HBsAg, hepatitis B surface antigen; HBV, hepatitis B virus; HCC, hepatocellular carcinoma; HCV, hepatitis C virus; HIV, human immunodeficiency virus; IFN, interferon; IQR, interquartile range; NA, nucleos(t)ide analogue; qHBsAg, quantitative HBsAg; SD, standard deviation; ULN, upper limit of normal.

## Supplementary Table 7: Characteristics of the study population by the presence of quantitative and semi-quantitative HBsAg data, among centres routinely collecting HBsAg data (Centres 1 and 2)

| **Variables** | **Complete qHBsAg and semi-quantitative HBsAg data (n=862)** | | **Missing qHBsAg and semi-quantitative HBsAg data (n=233)** | |
| --- | --- | --- | --- | --- |
|  | **n (%)** | **95% CI** | **n (%)** | **95% CI** |
| **Male sex** | 476 (55.2) | 51.9–58.5 | 111 (47.6) | 41.2–54.1 |
| **Age, years** |  | |  | |
| Mean (SD) | 44.5 (12.1) | | 45.2 (14.2) | |
| Median (IQR) | 42.0 (35.0–51.0) | | 44.0 (35.0–55.0) | |
| **Age group, years** |  |  |  |  |
| <25 | 12 (1.4) | 0.6–2.2 | 11 (4.7) | 2.0–7.4 |
| 25–49 | 602 (69.8) | 66.8–72.9 | 141 (60.5) | 54.2–66.8 |
| 50–64 | 184 (21.3) | 18.6–24.1 | 58 (24.9) | 19.3–30.4 |
| ≥65 | 64 (7.4) | 5.7–9.2 | 23 (9.9) | 6.0–13.7 |
| **Ethnicity^†^** |  |  |  |  |
| White | 234 (27.1) | 24.2–30.1 | 57 (24.5) | 18.9–30.0 |
| Mixed | 28 (3.2) | 2.1–4.4 | 14 (6.0) | 3.0–9.1 |
| Asian | 240 (27.8) | 24.8–30.8 | 60 (25.8) | 20.1–31.4 |
| Black | 131 (15.2) | 12.8–17.6 | 63 (27.0) | 21.3–32.7 |
| Other/unknown | 229 (26.6) | 23.6–29.5 | 39 (16.7) | 11.9–21.5 |
| **Co-infections (HIV and/or HCV)** | 15 (1.7) | 0.8–2.6 | 10 (4.3) | 1.7–6.9 |
| **Hepatitis delta** | 5 (0.6) | 0.2–1.4 | 1 (0.4) | 0–2.4 |
| **Liver fibrosis** | 30 (3.5) | 2.3–4.7 | 6 (2.6) | 1.0–5.6 |
| **Cirrhosis (any history)** |  |  |  |  |
| Compensated | 34 (3.9) | 2.6–5.2 | 9 (3.9) | 1.8–7.2 |
| Decompensated cirrhosis | 12 (1.4) | 0.6–2.2 | 2 (0.9) | 0.1–3.1 |
| **HCC** | 6 (0.7) | 0.3–1.5 | 0 |  |
| **CKD** | 39 (4.5) | 3.1–5.9 | 12 (5.2) | 2.3–8.0 |
| **HBeAg status** |  |  |  |  |
| Positive | 59 (8.5) | 6.4–10.6 | 21 (10.7) | 6.4–15.0 |
| Negative | 635 (91.5) | 89.4–93.6 | 176 (89.3) | 85.0–93.6 |
| Missing | 168 (19.5) | 16.8–22.1 | 36 (15.5) | 10.8–20.1 |
| **HBV DNA, IU/mL** |  |  |  |  |
| Undetectable | 177 (21.5) | 18.7–24.3 | 29 (12.7) | 8.4–17.0 |
| Detectable–<2000 | 576 (69.8) | 66.7–73.0 | 165 (72.4) | 66.6–78.2 |
| 2000–<20,000 | 53 (6.4) | 4.8–8.1 | 23 (10.1) | 6.2–14.0 |
| ≥20,000 | 19 (2.3) | 1.3–3.3 | 11 (4.8) | 2.0–7.6 |
| Missing | 37 (4.3) | 2.9–5.6 | 5 (2.1) | 0.7–4.9 |
| **ALT** |  |  |  |  |
| ≤ULN | 752 (88.1) | 85.9–90.2 | 198 (90.4) | 86.5–94.3 |
| 1–<2 × ULN | 85 (10.0) | 7.9–12 | 17 (7.8) | 4.2–11.3 |
| 2–<5 × ULN | 11 (1.3) | 0.5–2.0 | 4 (1.8) | 0.5–4.6 |
| ≥5 × ULN | 6 (0.7) | 0.3–1.5 | 0 |  |
| Missing | 8 (0.9) | 0.4–1.8 | 14 (6.0) | 3.0–9.1 |
| **Treatment** |  |  |  |  |
| NA monotherapy | 322 (37.4) | 34.1–40.6 | 61 (26.2) | 20.5–31.8 |
| NA combination therapy | 18 (2.1) | 1.1–3.0 | 1 (0.4) | 0–2.4 |
| Prior Interferon Use | 0 |  | 0 |  |
| No treatment | 522 (60.6) | 57.3–63.8 | 171 (73.4) | 67.7–79.1 |

The table shows descriptive baseline data only; no formal hypothesis testing was conducted.

†White = ^’^White – British’, ‘White – Irish’, ‘White - any other White background’; Asian = ‘Asian or Asian British – Indian Asian or Asian British – Pakistani’, ‘Asian or Asian British – Bangladeshi’, ‘Asian or Asian British – Any other Asian background’, ‘Chinese’; Black = ‘Black or Black British – Caribbean’, ‘Black or Black British – African’, ‘Black or Black British – any other Black background’; Mixed = ‘Mixed – White and Black Caribbean’, ‘Mixed – White and Black African’, ’Mixed – White and Asian/Mixed – Any other mixed background’; Other = ‘Any other ethnic group’. Ethnicity was self-reported by individuals and recorded using standard national codes in the UK.
ALT, alanine aminotransferase; CI, confidence interval; CKD, chronic kidney disease; HBeAg, hepatitis B e antigen; HBsAg, hepatitis B surface antigen; HBV, hepatitis B virus; HCC, hepatocellular carcinoma; HCV, hepatitis C virus; HIV, human immunodeficiency virus; IQR, interquartile range; NA, nucleos(t)ide analogue; qHBsAg, quantitative HBsAg; SD, standard deviation; ULN, upper limit of normal.

## Supplementary Table 8: Characteristics of the Centre 2 study cohort by semi-quantitative HBsAg levels (S/CO)

|  | **Semi-quantitative HBsAg level** | | | | | | | | | |  |  |
| --- | --- | --- | --- | --- | --- | --- | --- | --- | --- | --- | --- | --- |
|  | **Granular stratification** | | | | | | **Broad stratification** | | | |  |  |
|  | **<100** | | **100–3965^†^** | | **>3965^†^** | | **<3965^†^** | | **>3965^†^** | | **Missing** | |
|  | **(n=60)** | | **(n=240)** | | **(n=299)** | | **(n=300)** | | **(n=299)** | | **(n=39)** | |
|  | **n (%)** | **95% CI** | **n (%)** | **95% CI** | **n (%)** | **95% CI** | **n (%)** | **95% CI** | **n (%)** | **95% CI** | **n (%)** | **95% CI** |
| **Male sex** | 35 (58.3) | 45.9–70.8 | 143 (59.6) | 53.4–65.8 | 174 (58.2) | 52.6–63.8 | 178 (59.3) | 53.8–64.9 | 174 (58.2) | 52.6–63.8 | 25 (64.1) | 49.0–79.2 |
| **Age, mean (SD)** | 49.8 (12.5) |  | 41.7 (11.8) |  | 43.1 (10.8) |  | 43.3 (12.4) |  | 43.1 (10.8) |  | 44.8 (12.8) |  |
| **Age group, years** |  |  |  |  |  |  |  |  |  |  |  |  |
| <50 | 32 (53.3) | 40.7–66.0 | 194 (80.8) | 75.9–85.8 | 226 (75.6) | 70.7–80.5 | 226 (75.3) | 70.5–80.2 | 226 (75.6) | 70.7–80.5 | 26 (66.7) | 51.9–81.5 |
| 50–64 | 18 (30.0) | 18.4–41.6 | 33 (13.8) | 9.4–18.1 | 62 (20.7) | 16.1–25.3 | 51 (17.0) | 12.7–21.3 | 62 (20.7) | 16.1–25.3 | 9 (23.1) | 11.1–39.3 |
| ≥65 | 10 (16.7) | 7.2–26.1 | 13 (5.4) | 2.6–8.3 | 11 (3.7) | 1.5–5.8 | 23 (7.7) | 4.7–10.7 | 11 (3.7) | 1.5–5.8 | 4 (10.3) | 2.9–24.2 |
| **Ethnicity^†^** |  |  |  |  |  |  |  |  |  |  |  |  |
| White | 12 (20.0) | 9.9–30.1 | 66 (27.5) | 21.9–33.1 | 85 (28.4) | 23.3–33.5 | 78 (26.0) | 21–31 | 85 (28.4) | 23.3–33.5 | 9 (23.1) | 11.1–39.3 |
| Mixed | 4 (6.7) | 1.8–16.2 | 11 (4.6) | 1.9–7.2 | 12 (4.0) | 1.8–6.2 | 12 (4.0) | 1.8–6.2 | 12 (4.0) | 1.8–6.2 | 1 (2.6) | 0.1–13.5 |
| Asian | 26 (43.3) | 30.8–55.9 | 54 (22.5) | 17.2–27.8 | 72 (24.1) | 19.2–28.9 | 80 (26.7) | 21.7–31.7 | 72 (24.1) | 19.2–28.9 | 11 (28.2) | 14.1–42.3 |
| Black | 1 (1.7) | 0–8.9 | 34 (14.2) | 9.8–18.6 | 38 (12.7) | 8.9–16.5 | 38 (12.7) | 8.9–16.4 | 38 (12.7) | 8.9–16.5 | 11 (28.2) | 14.1–42.3 |
| Other / Unknown | 17 (28.3) | 16.9–39.7 | 75 (31.2) | 25.4–37.1 | 92 (30.8) | 25.5–36 | 92 (30.7) | 25.4–35.9 | 92 (30.8) | 25.5–36.0 | 7 (17.9) | 7.5–33.5 |
| **Co-infections (HIV and or HCV)** | 0 |  | 6 (2.5) | 0.9–5.4 | 8 (2.7) | 1.2–5.2 | 6 (2.0) | 0.7–4.3 | 8 (2.7) | 1.2–5.2 | 6 (15.4) | 5.9–30.5 |
| **Hepatitis Delta** | 0 |  | 4 (1.7) | 0.5–4.2 | 4 (1.3) | 0.4–3.4 | 3 (1.0) | 0.2–2.9 | 4 (1.3) | 0.4–3.4 | 0 |  |
| **Liver fibrosis** | 4 (6.7) | 1.8–16.2 | 7 (2.9) | 1.2–5.9 | 8 (2.7) | 1.2–5.2 | 8 (2.7) | 1.2–5.2 | 8 (2.7) | 1.2–5.2 | 0 |  |
| **Compensated cirrhosis** | 2 (3.3) | 0.4–11.5 | 7 (2.9) | 1.2–5.9 | 13 (4.3) | 2.0–6.7 | 8 (2.7) | 1.2–5.2 | 13 (4.3) | 2.0–6.7 | 3 (7.7) | 1.6–20.9 |
| **Decompensated cirrhosis** | 1 (1.7) | 0–8.9 | 3 (1.2) | 0.3–3.6 | 5 (1.7) | 0.5–3.9 | 7 (2.3) | 0.9–4.7 | 5 (1.7) | 0.5–3.9 | 4 (10.3) | 2.9–24.2 |
| **HCC (any history)** | 0 |  | 4 (1.7) | 0.5–4.2 | 5 (1.7) | 0.5–3.9 | 3 (1.0) | 0.2–2.9 | 5 (1.7) | 0.5–3.9 | 0 |  |
| **CKD** | 3 (5.0) | 1.0–13.9 | 10 (4.2) | 1.6–6.7 | 10 (3.3) | 1.3–5.4 | 13 (4.3) | 2.0–6.6 | 10 (3.3) | 1.3–5.4 | 1 (2.6) | 0.1–13.5 |
| **qHBsAg (S/CO value)** |  |  |  |  |  |  |  |  |  |  |  |  |
| Mean (SD) | 23.1 (25.1) | | 2563.3 (1143.7) | | 5023.9 (1500.2) | | 2055.3 (1442.8) | | 5023.9 (1500.2) | |  |  |
| Median (IQR) | 13.3 (3.4–35.2) | | 2866.8 (1939.7–3466.2) | | 4930.2 (4460.2–5333.1) | | 2479.5 (377.1–3369.7) | | 4930.2 (4460.2–5333.1) | |  |  |
| **HBeAg status** |  |  |  |  |  |  |  |  |  |  |  |  |
| Positive | 3 (7.0) | 1.5–19.1 | 24 (12.6) | 7.9–17.3 | 19 (8.3) | 4.7–11.8 | 25 (10.8) | 6.8–14.8 | 19 (8.3) | 4.7–11.8 | 4 (30.8) | 9.1–61.4 |
| Negative | 40 (93.0) | 80.9–98.5 | 167 (87.4) | 82.7–92.1 | 211 (91.7) | 88.2–95.3 | 207 (89.2) | 85.2–93.2 | 211 (91.7) | 88.2–95.3 | 9 (69.2) | 38.6–90.9 |
| Missing | 17 (28.3) | 16.9–39.7 | 49 (20.4) | 15.3–25.5 | 69 (23.1) | 18.3–27.9 | 68 (22.7) | 17.9–27.4 | 69 (23.1) | 18.3–27.9 | 26 (66.7) | 51.9–81.5 |
| **HBV DNA levels** |  |  |  |  |  |  |  |  |  |  |  |  |
| Undetectable | 12 (20.7) | 10.3–31.1 | 34 (15.2) | 10.5–19.9 | 55 (19.6) | 14.9–24.2 | 45 (16.0) | 11.7–20.3 | 56 (19.9) | 15.3–24.6 | 0 |  |
| Detectable–<2000 | 46 (79.3) | 68.9–89.7 | 177 (79.0) | 73.7–84.4 | 216 (76.9) | 71.9–81.8 | 223 (79.4) | 74.6–84.1 | 216 (76.9) | 71.9–81.8 | 36 (94.7) | 82.3–99.4 |
| 2000–<20,000 | 0 |  | 7 (3.1) | 1.3–6.3 | 7 (2.5) | 1.0–5.1 | 7 (2.5) | 1.0–5.1 | 7 (2.5) | 1.0–5.1 | 2 (5.3) | 0.6–17.7 |
| ≥20,000 | 0 |  | 6 (2.7) | 1.0–5.7 | 3 (1.1) | 0.2–3.1 | 6 (2.1) | 0.8–4.6 | 2 (0.7) | 0.1–2.5 | 0 |  |
| Missing | 2 (3.3) | 0.4–11.5 | 16 (6.7) | 3.5–9.8 | 18 (6.0) | 3.3–8.7 | 19 (6.3) | 3.6–9.1 | 18 (6.0) | 3.3–8.7 | 1 (2.6) | 0.1–13.5 |
| **ALT** |  |  |  |  |  |  |  |  |  |  |  |  |
| ≤ULN | 52 (86.7) | 75.4–94.1 | 195 (81.6) | 76.7–86.5 | 268 (90.8) | 87.6–94.1 | 247 (82.9) | 78.6–87.2 | 268 (89.9) | 86.5–93.3 | 32 (100) | 89.1–100 |
| 1–<2 × ULN | 8 (13.3) | 5.9–24.6 | 37 (15.5) | 10.9–20.1 | 24 (8.1) | 5.0–11.3 | 45 (15.1) | 11.0–19.2 | 27 (9.1) | 5.8–12.3 | 0 |  |
| 2–<5 × ULN | 0 |  | 5 (2.1) | 0.7–4.8 | 2 (0.7) | 0.1–2.4 | 5 (1.7) | 0.5–3.9 | 1 (0.3) | 0–1.9 | 0 |  |
| ≥5 × ULN | 0 |  | 2 (0.8) | 0.1–3.0 | 1 (0.3) | 0–1.9 | 1 (0.3) | 0–1.9 | 2 (0.7) | 0.1–2.4 | 0 |  |
| Missing | 0 |  | 1 (0.4) | 0–2.3 | 4 (1.3) | 0.4–3.4 | 2 (0.7) | 0.1–2.4 | 1 (0.3) | 0–1.8 | 7 (17.9) | 7.5–33.5 |
| **Treatment** |  |  |  |  |  |  |  |  |  |  |  |  |
| NA monotherapy | 5 (8.3) | 2.8–18.4 | 58 (24.2) | 18.8–29.6 | 110 (36.8) | 31.3–42.3 | 65 (21.7) | 17.0–26.3 | 107 (35.8) | 30.4–41.2 | 6 (15.4) | 5.9–30.5 |
| NA combination therapy | 4 (6.7) | 1.8–16.2 | 11 (4.6) | 1.9–7.2 | 5 (1.7) | 0.5–3.9 | 13 (4.3) | 2.0–6.6 | 8 (2.7) | 1.2–5.2 | 3 (7.7) | 1.6–20.9 |
| Prior Interferon use | 0 |  | 0 |  | 0 |  | 0 |  | 0 |  | 0 |  |
| No treatment | 51 (85.0) | 73.4–92.9 | 171 (71.2) | 65.5–77.0 | 184 (61.5) | 56.0–67.1 | 222 (74.0) | 69.0–79.0 | 184 (61.5) | 56.0–67.1 | 30 (76.9) | 60.7–88.9 |

The table shows descriptive baseline data only; no formal hypothesis testing was conducted.

†3965 is the median of semi-quantitative HBsAg levels (S/CO values) from Centre 2; ‡White = ^’^White – British’, ‘White – Irish’, ‘White - any other White background’; Asian = ‘Asian or Asian British – Indian Asian or Asian British – Pakistani’, ‘Asian or Asian British – Bangladeshi’, ‘Asian or Asian British – Any other Asian background’, ‘Chinese’; Black = ‘Black or Black British – Caribbean’, ‘Black or Black British – African’, ‘Black or Black British – any other Black background’; Mixed = ‘Mixed – White and Black Caribbean’, ‘Mixed – White and Black African’, ’Mixed – White and Asian/Mixed – Any other mixed background’; Other = ‘Any other ethnic group’. Ethnicity was self-reported by individuals and recorded using standard national codes in the UK.
ALT, alanine aminotransferase; CI, confidence interval; CKD, chronic kidney disease; HBeAg, hepatitis B e antigen; HBsAg, hepatitis B surface antigen; HBV, hepatitis B virus; HCC, hepatocellular carcinoma; HCV, hepatitis C virus; HIV, human immunodeficiency virus; qHBsAg, quantitative HBsAg; S/CO, signal-to-cutoff ratio; SD, standard deviation; ULN, upper limit of normal.
